# Self-supervised non-dominated sorted model for co-clustering

**DOI:** 10.1038/s41598-026-42498-9

**Published:** 2026-03-04

**Authors:** Xu Li, Hongjun Wang, Wuchun Yang, Luqing Wang, Tianrui Li

**Affiliations:** 1 School of Computing and Artificial Intelligence, Southwest Jiaotong University, Chengdu, 610000 China; 2 Engineering Research Center of Sustainable Urban Intelligent Transportation, Ministry of Education, Chengdu, 610000 China

**Keywords:** Co-clustering, Multi-objective optimization, Self-supervised, Genetic algorithm, Non-dominated sorting, Computational science, Computer science

## Abstract

Co-clustering is widely used for data analysis that independently reveals the clustering structures of rows and columns while also identifying their inter-relationships, which renders it more informative than conventional one-way clustering methods. Co-clustering is to not only cluster the samples and features of original data, but also mine the relationship between samples and features, and this is naturally a multi-objective problem. However, researchers frequently utilize the method of single-objective optimization to solve the co-clustering issue, while disregarding its multi-objective nature, and the side information in the original data is also ignored. To address these problems, we propose a self-supervised non-dominated sorted model for co-clustering (SNSC), which is represented by a group of multi-objective functions. The model not only perfectly aligns with the multi-objective nature of co-clustering tasks but also utilizes the supervised information in the original data. The objective function group consists of four objective functions acting on the original data and similarity matrix respectively. The heuristic initialization method with self-supervised properties is used in conjunction with the random initialization method, which improves the efficiency of the model and reduces the likelihood of converging to local optima. The overall model remains unsupervised, as all the supervised information is derived from the original data. Further, the algorithm for the SNSC model is designed by using the idea of the genetic algorithm, which is theoretically supported, and the complexity analysis of the algorithm is given. Finally, experiments on 12 datasets and 5 comparison algorithms show that the SNSC algorithm has significant advantages.

## Introduction

As an unsupervised machine learning method, co-clustering can analyze objects and their features at the same time^1^. Clustering is more efficient when all columns contributes to the similarity between rows, while co-clustering is superior when the similarity is relevant to just a select group of columns. In contrast to clustering, co-clustering can explore many new and valuable data patterns, so it is widely used in many real-world applications, such as document clustering^2^, recommendation systems^3^ and image segmentation^4,5^.

Co-clustering can often be formulated as an optimization problem^6^. Numerous researchers have investigated a variety of optimization methods to solve problems across different domains, such as genetic algorithms(GA)^7^, particle swarm optimization algorithms^8^, differential evolution algorithms^9^, chaotic soil wolf optimization algorithms^10^, and improved multidimensional red fox optimization algorithms^11^. Among these, genetic algorithms are recognized as a crucial branch due to their scalability and potential for global optimization^12,13^. Moreover, a wealth of literature^14^ has strongly verified the effectiveness of the genetic algorithm in optimization tasks, especially for clustering tasks^8,15^.

However, most existing co-clustering methods tend to formulate the problem as a single-objective optimization task, which inevitably oversimplifies the inherent complexity of co-clustering^16–18^. These methods typically combine row-distance-based and column-clustering-based objectives using fixed weighting parameters, which complicates the determination of optimal weight distributions across different application scenarios. Moreover, many of these methods fail to fully exploit the supervisory information embedded in the data, limiting their ability to capture rich and meaningful data structures^19–23^. This issue is particularly evident in unsupervised co-clustering approaches, which often lack effective mechanisms to incorporate external knowledge to guide the optimization process.

To address the inherent multi-objective attri-butes of co-clustering tasks, the common approach merges row-distance-based and column-clustering-based objectives using weighted parameters for single-objective optimization. However, identifying suitable weight parameters is a significant challenge, and the weight distribution might vary across different application scenarios. Furthermore, unsupervised clustering underutilizes original data and lacks supervisory guidance during optimization^24^. To tackle these challenges, we leverage self-supervised learning to devise a hybrid initialization approach that combines a heuristic initialization method with self-supervised properties and a random initialization method in this paper. Additionally, it adopts four interconnected objective formulas that form a composite goal. An enhanced non-dominated sorting genetic algorithm is utilized for resolution. The proposed algorithm is termed the Self-supervised Non-dominated Sorted Model for Co-clustering. There are three contributions of this paper.

- The co-clustering model and the similarity learning model are integrated to design four objective functions and combine the objective functions into a unified function group for co-clustering.
- A multi-objective co-clustering model, specially customized for the proposed group of objective functions, is designed.
- The Hybrid-initialization method that merges heuristic self-supervised initialization with random initialization techniques is implemented to improve the robustness and performance of the model.

The organization of the remainder of this paper is as follows. In Section Relate work, the related existing work is presented. In Section Proposed model, a detailed illustration of the proposed SNSC is provided. In Section Experiment Design and Result Analysis, the evaluation methods and the experimental results are reported. Finally, the conclusion and future work of this paper are provided in Section Conclusion.

## Relate work

### Co-clustering

Hartigan^25^ first proposed the concept of co-clustering, also known as block clustering. Co-clustering is expected to divide the data matrix into interrelated blocks, and the objective function of it is defined as the sum of the variance of each block. Subsequently, Cheng^26^ conducted a highly representative work and introduced a more effective objective function, namely the mean square residual. Since then, co-clustering has rapidly risen in popularity and drawn growing interest from both the theoretical and practical research sectors. It has been proven that co-clustering can explore potential data patterns and improve clustering results compared to unilateral clustering^27,28^. Benefiting from this feature, co-clustering is versatile across a wide range of real-world applications, such as community detection^29,30^, discovering mobility patterns^31^, and treatment behavior recognition^32^. Current co-clustering methodologies are broadly classified into six categories, based on fuzzy theory^2,33^, based on graph theory^34^, based on information theory^16,35^, based on probability theory^36^, based on matrix factorization^37^, and other types^8,38^.

The Fuzzy Clustering for Categorical Multivariate Data (FCCM)^39^ first introduced fuzzy theory into the co-clustering problem, aiming to minimize the sum of distances from the cluster center to the data points. Then, Krishna^2^ introduced significant improvements to FCCM, enabling effective clustering of extensive text corpora. Presently, fuzzy-based co-clustering has seen considerable exploration, indicating its growing importance in this research area^3,37,40,41^.

The second approach is grounded in graph theory, such as co-clustering based on bipartite graph partitioning^42^ and co-clustering based on learning structured optimal bipartite graph partitioning^34^. It is effective to use bipartite graphs to solve multi-view problems^43,44^, as they can converge to the ideal value faster. Zhu^5^ proposed a more expressive method than classical hypergraphs, using edge-dependent vertex weights to co-cluster vertices and hyperedges of hypergraphs. Additionally, the use of bipartite graph partitioning methods has been shown to significantly enhance the performance and scalability of clustering algorithms, especially in high-dimensional datasets^45^.

The third approach is based on information theory. The Information-theoretic Co-clustering (ITCC)^35^ method enhances clustering quality by optimizing mutual information, essentially minimizing the variance of mutual information. Banerjee proposed the Bregman co-clustering algorithm^16^, which refines the information-theoretic framework for greater universality. Following the principle of ITCC, Clemens Blöchl^46^ formulated an information-theoretic cost function that integrates graph theory with information theory, facilitating effective co-clustering of bipartite graphs.

The fourth approach is based on probability theory, with the Bayesian Co-clustering (BCC) model^36^ and the Gaussian Co-clustering model^47^ being notably classic examples. The BCC model is particularly valued for its scalability, inspiring researchers to adapt it for various applications. For instance, Hansen introduced a nonparametric version of the BCC model^48^ to facilitate co-clustering on large networks, while Du enhanced the BCC framework for deployment in mobile crowd-sensing systems^49^.

The fifth approach utilizes non-negative matrix factorization (NMF) technology, renowned for its efficacy in clustering^50^ and co-clustering. Various NMF-based co-clustering methods have emerged due to this technology’s robust performance. Zhao Li introduced a weighted NMF algorithm to enhance spectral co-clustering results^37^. The Neighbor Constrained Co-clustering (SNCC)^17^ optimizes clustering by ensuring samples and their neighbors share the same row cluster. Moreover, the Dual Regularized Co-Clustering (DRCC)^18^ integrates NMF with graph regularization, accounting for the data’s geometric structure. Nonnegative Matrix Factorization with Adaptive Neighbors (NMFAN)^51^ adapts to learn the similarity matrix, enabling the construction of an appropriate graph for clustering. The Tri-regularized Nonnegative Matrix Tri-factorization(TRNMTF)^19^ model for co-clustering is proposed to eliminate noise in the model. In addition to the methods discussed, a plethora of exceptional studies utilizing diverse approaches have significantly contributed to the co-clustering domain^38,52,53^, each propelling the advancement of this field.

The sixth approach utilizes deep learning techniques, which have gained significant attention in recent years due to their ability to model complex patterns and hierarchies in data. Deep clustering methods, such as semi-supervised deep clustering, leverage neural networks to learn feature representations and optimize clustering simultaneously. One such method is SDAC-DA, which uses a dual-autoencoder framework to learn node embeddings that are more suitable for clustering and enhances the overall clustering process in an end-to-end fashion^54^. These deep learning-based approaches have demonstrated substantial improvements in clustering performance, especially for attributed graphs with semi-supervised labels. Furthermore, a deep learning-based co-clustering method, integrates deep neural networks with co-clustering techniques to jointly learn row and column representations in a data matrix, which leads to better clustering outcomes^23^.

In summary, co-clustering has evolved into a powerful technique with various approaches rooted in different theories, Furthermore, a recent approach in co-clustering leverages entropy-regularized optimal transport to simultaneously cluster instances and features by optimizing the coupling matrix between them, thus providing an efficient and theoretically grounded framework for co-clustering^20^. Each approach has demonstrated its effectiveness in tackling clustering challenges, especially with high-dimensional or sparse data. Recent advancements have brought deep learning techniques to the forefront, offering promising solutions in semi-supervised clustering and improving clustering performance for attributed graphs. However, despite its effectiveness, challenges remain in the scalability and efficiency of co-clustering methods, particularly for large datasets. Biernacki et al.^55^ provide an in-depth look at model-based co-clustering and the challenges of high-dimensional data. Wang et al.^56^ offer a broad survey of co-clustering methods, highlighting key developments and future trends. Battaglia et al.^57^ review the main methods and recent advancements in co-clustering, along with open problems and promising research directions. These surveys offer a valuable resource for both novice and expert researchers, providing a solid foundation for further exploration in the field.

### Genetic algorithm

Introduced by Holland in the 1960s and 1970 s, the genetic algorithm, a fundamental multi-objective optimization tool, draws on evolution’s natural selection and survival mechanisms^58^. Schaffer advanced the field with the first genetic algorithm in 1985^59^. Multi-objective genetic algorithms are categorized into two types. One that combines multiple objectives into a unified function for optimization, and another that aims for a Pareto optimal solution set. Hajela enriched the genetic algorithm with a weighting approach, pioneering a weight-based method for the first type of multi-objective optimization in 1992^60^.

The second type of multi-objective optimization algorithms is divided into two categories according to the presence or absence of an elitist retention strategy. Initially, algorithms like Fonseca’s multi-objective genetic algorithm lacked this strategy^61^. Horn integrated niche technology into genetic algorithms in 1994, introducing niche Pareto genetic algorithms^62^. Simultaneously, Srinivas incorporated non-dominated sorting into the genetic algorithm’s search and selection mechanisms, establishing the pioneering Nondominated Sorting Genetic Algorithm (NSGA)^63^.

Building on this momentum, Zitzler integrated the elitist strategy into genetic algorithms and created the Strength Pareto Evolutionary Algorithm(SPEA)^64^ in 1999. Subsequently, the SPEA was further strengthened^65^. Concurrently, Corne introduced a supergrid technique to enhance population selection and diversity maintenance in genetic algorithms, proposing the Pareto Envelope-based Selection Algorithm(PESA)^66^ and its enhancement PESA-2^67^. Coello developed a micro-genetic algorithm (micro-GA)in 2001, which utilizes the repeated initialization of a small number of populations^68^.

Following these developments, Deb introduced a fast non-dominated sorting algorithm and proposed NSGA-II^69^ in 2002. This algorithm remains a focal point in multi-objective optimization research and was further evolved into NSGA-III by Deb and Jain addressing the non-dominated sorting issue in super-multi-objective settings^70,71^. Cheng and colleagues proposed improvements to NSGA-III in 2016, which utilized reference vectors to guide multi-objective optimization^72^. Liu further improved the convergence of NSGA-III by incorporating the concepts of the *k*-means algorithm in 2019^73^.

By 2021, Tian proposed a constrained multi-objective co-evolutionary framework to tackle constrained multi-objective optimization problems in smaller feasible regions^74^. Cai developed a network-based inverse generational distance index to assess the approximate convergence and diversity of Pareto frontiers in multi-objective optimization^75^. Hao fused the ideas of hyper-heuristics and evolutionary algorithms to propose a framework for graph-based hyper-heuristic multi-objective evolutionary algorithms in 2021^76^. Moreover, numerous studies utilizing multi-objective optimization algorithms have significantly contributed to advancing the field^15,77,78^.

## Proposed model

**Fig. 1:**
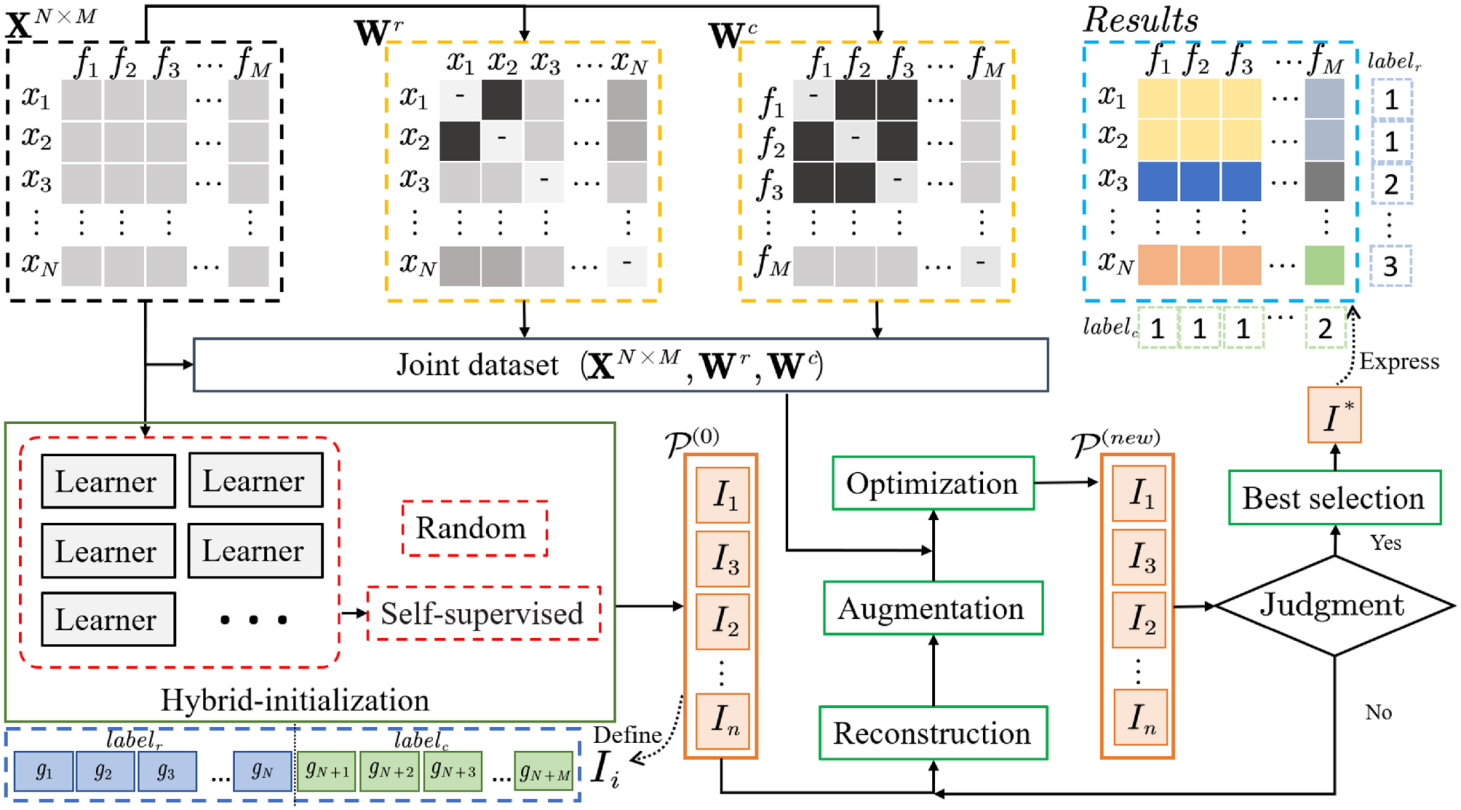
model of SNSC. The dataset $\textbf{X}^{N\times M}$ serves as the input for the model, with $x_i$ denoting an individual sample and $f_i$ representing a specific feature. The matrices $\textbf{W}^r$ and $\textbf{W}^c$, highlighted by yellow dotted lines in the top of the figure, is the sample and feature connection matrices, respectively. Their detailed explanations are forthcoming in Subsections Preliminaries. Additionally, the multi-objective function group designed for the SNSC model is discussed in Subsection Self-supervised Non-dominated Sorted Model for Co-clustering. The function group is utilized on the joint dataset is formed by $\textbf{X}^{N\times M}$, $\textbf{W}^r$, and $\textbf{W}^c$. The inference process of the SNSC model, depicted in the green box, aligns with the discussions in Subsection Inference of SNSC. Notably, this section integrates multiple learners within the Hybrid-initialization module to acquire self-supervised information. The definition of *I* and the significance of the result $I^*$ are in the blue box in the figure, wherein $label_r$ and $label_c$ correspond to the clustering outcomes for samples and features, respectively.

The Self-supervised Non-dominated Sorted Model for Co-clustering is described in detail in this section. First some preparations for the model are presented, then the group of objective functions for the model is detailed, followed immediately by a discussion of the model’s inference process, and finally, the algorithm corresponding to the model is proposed and its time complexity is analyzed. The overall framework of the SNSC model is shown in Fig.1.

### Preliminaries

This subsection describes the process of constructing similarity information. To optimize clustering quality, self-supervised learning is employed, which serves as a key component of the co-clustering approach. Self-supervised learning enables the model to learn meaningful data representations without relying on explicit labels. In this approach, the model leverages the inherent similarity relationships between data points, using only the data itself to optimize clustering quality.

This approach enables the model to learn from the data structure, rather than relying on external supervision or manually labeled data. This is especially beneficial in co-clustering tasks, where both rows (samples) and columns (features) must be clustered simultaneously. By incorporating both global and local similarity information, self-supervised learning enhances the clustering process, something traditional methods relying solely on unsupervised optimization cannot achieve^79^.

For example, the sample similarity information $\textbf{W}^{r}$ is calculated based on the distance $||x_i - x_j||$ between row data $x_i$ and $x_j$ in the dataset $\textbf{X}$. This similarity information then guides the clustering process by ensuring that similar samples are grouped together. Unlike traditional methods that rely on heuristic approaches or fixed parameters, our self-supervised approach dynamically adjusts clustering boundaries based on the data’s internal structure, leading to improved clustering accuracy.

While traditional clustering techniques like *k*-means^80^ focus primarily on distance measures between points, our self-supervised approach integrates both global and local similarity information. This improves clustering by enhancing compactness within clusters and promoting separation between clusters, thus leveraging the inherent structure of the data.

In the SNSC model, the similarity information is divided into two aspects: sample similarity information $\textbf{W}^{r}$ and feature similarity information $\textbf{W}^{c}$. The construction process for both is similar, so the subsequent content uses sample data as an example to explain the construction process.

Sample similarity information construction is divided into two steps, which are global sample similarity $\textbf{W}^{r1}$ and local sample similarity $\textbf{W}^{r2}$. As shown in Fig.1, although $\textbf{W}^{r}$ and $\textbf{W}^{c}$ are not data in the form of labels, but the similarity relationship between their objects, they directly obtain and supervise the calculation process through the original data.

The sample similarity information $\textbf{W}^{r}$ is calculated from the distance $||x_i - x_j||$ between the row data $x_i$ and $x_j$ in the dataset $\textbf{X}$, where $\textbf{W}^{r1}_{i,:}$ portrays the similarity between any two samples, which emphasizes the connectivity of $x_i$ in the global space, and $\textbf{W}^{r2}_{i,:}$ portrays the similarity between the sample $x_i$ and its nearby strongly correlated samples, which emphasizes the connectivity of the sample $x_i$ in its adjacent local space. $\textbf{W}^{r1}$ is calculated by

1 $$\begin{aligned} W^{r1}_{ij}= \exp {\left( \frac{-||x_i-x_j||_2^2}{2\sigma ^r_i\sigma ^r_j} \right) }, \end{aligned}$$

the closer between $x_i$ and $x_j$, the smaller $||x_i-x_j||_2^2$ is, which means that the two samples are more similar and their corresponding $W^{r1}_{ij}$ value is larger. $\sigma ^r$ is a sensitive parameter, as the larger its value, the faster $W^{r1}_{ij}$ decays. Therefore, to better reflect the true distribution among samples^81^, we calculate the corresponding $\sigma ^r_i$ value for each sample using

2 $$\begin{aligned} \sigma ^r_i = \frac{1}{n^{k}_r}\sum _{i=1}^{n^{k}_r}||x_i-x_j||, \end{aligned}$$

where $\sigma ^r_i$ is determined by $x_i$ and the $n^{k}_r$ nearest samples to $x_i$. The local sample similarity $\textbf{W}^{r2}$ is calculated by

3 $$\begin{aligned} W^{r2}_{ij} = {\left\{ \begin{array}{ll} \exp {\left( \frac{-||x_i-x_j||_2^2}{2\sigma ^r_i\sigma ^r_j} \right) }, & a^{r}_{ij} = 1 \\ 0, & a^{r}_{ij}= 0 \end{array}\right. }, \end{aligned}$$

where $a^{r}_{ij}$ is indicates a connection between $x_i$ and $x_i$. When $a^{r}_{ij}=1$, it implies that $x_i$ and $x_i$ are the nearest neighbor of each other with parameter $n^{k}_r$. For all other cases, $a^{r}_{ij}=0$.

By incorporating both global and local similarity information, the resulting similarity sample information $\textbf{W}^{r}$ is

4 $$\begin{aligned} \textbf{W}^{r}= \textbf{W}^{r1}+\textbf{W}^{r2}. \end{aligned}$$

The feature similarity information $\textbf{W}^{c}$ can be obtained by the same method. It is noteworthy that the number of neighbors is determined by the specified neighbor parameter *Pk*. For a given dataset $\textbf{X}^{N\times M}$, in the process of calculating the row similarity $\textbf{W}^{r}$, $n^{k}_r = Pk \times N$, whereas for the column similarity $\textbf{W}^{c}$, $n^{k}_c = Pk \times M$.

The time complexity for constructing the sample similarity information in a dataset with $N$ samples and $M$ features is as follows. Calculating the global sample similarity $\textbf{W}^{r1}$ requires computing the pairwise distances between all samples, which results in a complexity of $O(N^2 \cdot M)$. The calculation of $\sigma ^r_i$ for each sample involves finding its nearest neighbors, leading to a complexity of $O(N^2 \cdot n^{k}_r \cdot M)$. Similarly, the local sample similarity $\textbf{W}^{r2}$ is computed with a complexity of $O(N^2 \cdot M)$. Merging the global and local similarity matrices has a complexity of $O(N^2)$. Finally, computing the feature similarity matrix $\textbf{W}^{c}$ incurs a complexity of $O(M^2 \cdot N)$. Therefore, the total time complexity for constructing the similarity information is $O(N^2 \cdot M + N^2 \cdot n^{k}_r \cdot M + M^2 \cdot N + N \cdot n^{k}_l \cdot M^2)$, where $N$ is the number of samples, $M$ is the number of features, and $n^{k}_r$ and $n^{k}_l$ is the number of nearest neighbors per sample and feature.

### Self-supervised non-dominated sorted model for co-clustering

The SNSC model addresses the co-clustering problem through four objective functions. Given the dataset $\textbf{X}^{N \times M}$, where *N* denotes the number of samples and *M* represents the number of features, the vector $x_i$ represents the *i*-th row in $\textbf{X}$, and $f_i$ denotes the *i*-th column vector. The SNSC model simultaneously operates on both the sample (rows) and feature (columns) directions of the dataset to achieve the co-clustering task. This process is illustrated in Fig.1 and can also be represented as follows:

5 $$\begin{aligned} \textbf{X} \xrightarrow {SNSC} I^*, \quad I^* = \left[ g_1, g_2, g_3, \dots , g_N, \dots , g_{N+M} \right] , \end{aligned}$$

where $I^*$ is the co-clustering result of the SNSC model, including sample labels ($[g_1, g_2, g_3, \dots , g_N]$) and feature labels ($[g_{N+1}, g_{N+2}, g_{N+3}, \dots , g_{N+M}]$).

The multi-objective function group for SNSC consists of four objective functions: the row clustering objective function, the column clustering objective function, the row self-supervised loss function, and the column self-supervised loss function. These functions are represented as:

6 $$\begin{aligned} I^* : \arg \min _I \varGamma (I) = \left[ \gamma _1(I), \gamma _2(I), \gamma _3(I), \gamma _4(I) \right] ^{T}, \end{aligned}$$

where *I* represents the co-clustering result of rows (samples in $\textbf{X}$) and columns (features in $\textbf{X}$), and is treated as an individual in the population within the SNSC model. $\gamma _1(I)$ and $\gamma _2(I)$ represent the ratio of intra-cluster compactness to inter-cluster dispersion for rows and columns, respectively, while $\gamma _3(I)$ and $\gamma _4(I)$ are the row and column self-supervised loss functions.

#### Row clustering objective function $\gamma _1(I)$

The row clustering objective function $\gamma _1(I)$ is designed to optimize the tightness of the samples within the same cluster while maximizing the dispersion between clusters. Specifically, the formula calculates the ratio of intra-cluster compactness to inter-cluster dispersion for the row clusters. It is defined as follows:

7 $$\begin{aligned} \gamma _1(I) = \frac{\sum _{i=1}^{k_r} \sum _{x_p \in \mathscr {R}_i} \Vert x_p - \mu _r^i \Vert ^2}{\sum _{i=1}^{k_r} \sum _{j=i+1}^{k_r} \Vert \mu _r^i - \mu _r^j \Vert ^2}, \end{aligned}$$

where $k_r$ is the number of row clusters, $\mathscr {R}_i$ is the *i*-th row cluster in *I*, $x_p$ is the sample, and $\mu _r^i$ is the center of the *i*-th row cluster.The numerator of this equation measures the compactness of the samples within each row cluster, while the denominator measures the separation between different row clusters. Minimizing this ratio leads to tighter clusters and larger separations between clusters, improving the clustering quality.

#### Column clustering objective function $\gamma _2(I)$

The column clustering objective function $\gamma _2(I)$ operates similarly to the row clustering function but is applied to the features. This objective function aims to ensure that similar features are grouped together, while maximizing the separation between feature clusters. It is defined as:

8 $$\begin{aligned} \gamma _2(I) = \frac{\sum _{i=1}^{k_c} \sum _{f_q \in \mathscr {C}_i} \Vert f_q - \mu _c^i \Vert ^2}{\sum _{i=1}^{k_c} \sum _{j=i+1}^{k_c} \Vert \mu _c^i - \mu _c^j \Vert ^2}, \end{aligned}$$

where $k_c$ is the number of column clusters, $\mathscr {C}_i$ is the *i*-th column cluster in *I*, $f_q$ is the feature, and $\mu _c^i$ is the center of the *i*-th column cluster.This formula mirrors $\gamma _1(I)$ but is applied to the columns, optimizing intra-cluster compactness and inter-cluster dispersion for the feature space. The optimization goal is to minimize the ratio of the intra-cluster tightness to inter-cluster separation for the columns, ensuring well-defined and well-separated feature clusters.

#### Row self-supervised loss function $\gamma _3(I)$

The row self-supervised loss function $\gamma _3(I)$ enforces the constraint that similar samples should be assigned to the same cluster. This function incorporates self-supervised learning by utilizing the similarity information in the dataset to guide the clustering process. It is defined as:

9 $$\begin{aligned} \gamma _3(I) = \sum _{i=1}^{N} \sum _{j=i+1}^{N} W_{ij}^{r} \Vert \epsilon _{i,:}^r - \epsilon _{j,:}^r \Vert ^2, \end{aligned}$$

where: $W_{ij}^r$ represents the row self-supervision information, which encodes the similarity between samples, $\epsilon ^r$ is the row effective segmentation matrix that contains sample group assignments, and $\epsilon ^r_{ij}$ is the self-supervised label.

The self-supervised label $\epsilon ^r_{ij}$ is defined as:

10 $$\begin{aligned} \epsilon ^r_{ij} = {\left\{ \begin{array}{ll} 1, & \text {if } q_i^r = g_j \\ 0, & \text {if } q_i^r \ne g_j \end{array}\right. }, \end{aligned}$$

where $q_i^r$ represents the cluster label of the *i*-th row cluster $\mathscr {R}_i$, $g_j$ represents the row cluster label corresponding to the *j*-th sample. Minimizing this function ensures that samples with high similarity, as indicated by the self-supervision matrix, are assigned to the same row cluster.

#### Column self-supervised loss function $\gamma _4(I)$

The column self-supervised loss function $\gamma _4(I)$ follows a similar structure to the row self-supervised loss function and is designed to ensure that similar features are clustered together. This function is defined as:

11 $$\begin{aligned} \gamma _4(I) = \sum _{i=1}^{M} \sum _{j=i+1}^{M} W_{ij}^c \Vert \epsilon _{i,:}^c - \epsilon _{j,:}^c \Vert ^2, \end{aligned}$$

where $W_{ij}^c$ represents the column self-supervision information, and $\epsilon ^c$ is the column effective segmentation matrix. This self-supervised loss function enforces that features with high similarity are grouped together, improving the column clustering process.

Finally, the objective function group for the SNSC model is obtained by combining Eq.7, Eq.8, Eq.9, and Eq.11. The co-clustering result improves as the values of the four objective functions decrease.

The row clustering objective function $\gamma _1(I)$ uses Euclidean distance to measure both the tightness of samples within the same cluster and the dispersion between samples in different clusters. However, the clustering process is further enhanced by the self-supervised loss function $\gamma _3(I)$. By leveraging the similarity information between samples, $\gamma _3(I)$ ensures that samples with high similarity are grouped together, which directly guides the row clustering process, helping to improve both the intra-cluster compactness and inter-cluster separation.

Similarly, the column clustering objective function $\gamma _2(I)$ works in tandem with the self-supervised loss function $\gamma _4(I)$, which ensures that similar features are grouped together, optimizing the clustering of features. $\gamma _2(I)$ measures the tightness and separation of feature clusters, while $\gamma _4(I)$ uses the self-supervised information to guide the feature clustering process, making sure that similar features are assigned to the same cluster.

In essence, the row and column clustering objectives ($\gamma _1(I)$ and $\gamma _2(I)$) focus on the overall structure of the data, while the self-supervised loss functions ($\gamma _3(I)$ and $\gamma _4(I)$) provide additional guidance by enforcing that similar samples and features are grouped together. The interaction between these objectives ensures that both the tightness within clusters and the separation between different clusters are optimized for both rows and columns.

The optimization process of this objective function group, as shown in Eq.6, is presented in the next subsection, which describes the process of minimizing the objective function group in detail. This combined optimization process ensures that the clustering is not only compact but also well-separated, leveraging both the inherent data structure and the self-supervised information to achieve high-quality co-clustering results.

### Inference of SNSC

The inference process of the model is accompanied by the design of operators such as hybrid-initialization, reconstruction, augmentation, optimization and best selection. In this section, each part is described and a brief demo of finding the final co-clustering results $I^*$ as shown in Fig.1.

#### Hybrid-initialization

In the inference process, it is first necessary to define the format of the calculation objects in the model, followed by an exposition of the hybrid-initialization method’s two components: random initialization and heuristic initialization. Notably, heuristic initialization acquires supervisory information via an unsupervised approach, thereby guiding the model’s optimization process. This mechanism epitomizes the self-supervised essence of the SNSC model, illustrating its ability to harness inherent data structures for guidance without relying on externally provided labels.

The inference object *I* is defined as a vector with the length of $N+M$, which as

12 $$\begin{aligned} \begin{aligned} I= & \left[ g_1,g_2,\cdots ,g_N,g_{N+1}, g_{N+2},\cdots ,g_{N+M} \right] . \end{aligned} \end{aligned}$$

For every *I*, the first *N* elements $label_r=[ g_1,g_2,\cdots ,g_N]$ represent a clustering result of samples in $\textbf{X}$, each element $g_i$ ($1\le i\le N$) is perceived as the cluster label for the sample $x_i$; the remaining *M* elements $label_c=[g_{N+1}, g_{N+2},\cdots ,g_{N+M}]$ represent a clustering result of features in $\textbf{X}$, where the value of each element $g_N+j (1\le j\le M)$ corresponds to the cluster label of the feature $f_j$ in $\textbf{X}$, as shown in the blue dotted box in the lower left corner of Fig.1.

After defining the format of *I*, additional initialization operations are carried out on the initial population $\mathscr {P}^{0}$, which comprises *I*. Firstly, initialize the population $\mathscr {P}^{(0)}$ to be of size *n* (i.e. $\mathscr {P}^{\left( 0 \right) }=\left\{ I_{1}^{\left( 0 \right) },I_{2}^{\left( 0 \right) },I_{3}^{\left( 0 \right) },\cdots ,I_{n}^{\left( 0 \right) }\right\}$), which indicate that the population $\mathscr {P}^{(0)}$ comprises *n* individuals.

Then, for the first ${\lfloor \frac{n}{2} \rfloor }$ individuals in $\mathscr {P}^{\left( 0 \right) }$, a bounded random number is utilized to populate element $g_i$ within each individual. For the initial *N* elements that represent sample cluster labels in $I^{(0)}_i$, a positive integer not exceeding $k_r$ is utilized to populate. For the remaining *M* elements that represent feature cluster labels, a positive integer not exceeding $k_c$ is utilized to populate, where $k_r$ and $k_c$ represent the number of sample clusters and feature clusters within dataset $\textbf{X}$, respectively.

Finally, for the remaining individual in $\mathscr {P}^{\left( 0 \right) }$, heuristic initialization methods with self-supervised nature will be used to populate the elements in individual $I^{(0)}_i$($i={(\lfloor \frac{n}{2}\rfloor +1):N+M}$), respectively. For a given dataset $\textbf{X}^{N\times M}$, a clustering algorithm(Learner in the red box in Fig.1 is randomly selected to perform a clustering operation on it to generate the first *N* elements and fill them; further, a clustering algorithm is randomly selected to perform a clustering operation on $\textbf{X}^{T}$ to generate the last *M* elements.

In hybrid-initialization, the random initialization part ensures the diversity of population $\mathscr {P}^{(0)}$, while the self-supervised initialization part ensures the convergence of the algorithm and the quality of the population. This initialization strategy aims to combine the advantages of randomness and guidance to improve the overall performance of the algorithm. Random initialization introduces diversity, helping the algorithm explore different regions of the solution space, and reducing the risk of falling into local optima. At the same time, the self-supervised initialization process guides the search process by leveraging the intrinsic structure of the data, promoting rapid convergence of the algorithm and increasing the probability of finding high-quality solutions.

Hybrid-initialization consists of random and heuristic initialization. For random initialization, the first $\lfloor \frac{n}{2} \rfloor$ individuals have $N + M$ labels assigned, with a time complexity of $O(n \cdot (N + M))$, assuming no clustering operations. For heuristic initialization, the remaining individuals also have $N + M$ labels assigned, with the same time complexity of $O(n \cdot (N + M))$, without clustering. Thus, the overall time complexity is $O(n \cdot (N + M))$.

#### Reconstruction of co-clustering results

The reconstruction of co-clustering results involves slicing and exchanging the information contained in two individuals to produce offspring with the same structure as their parents, similar to sexual reproduction in nature. Its purpose is to create more co-clustering results through the current population, increase population diversity, and avoid the algorithm falling into local optima. In situations of high population diversity, the reconstruction of co-clustering results can lead to a greater number of progeny individuals possessing diverse information from their progenitors, which is desirable.

**Algorithm 1:**
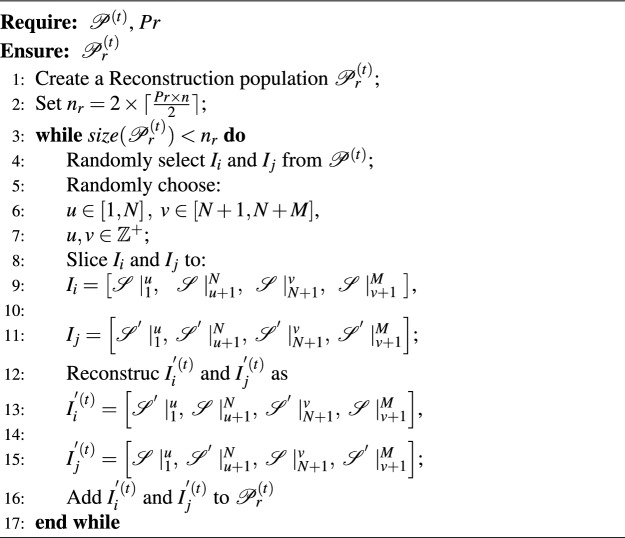
Reconstruction($$\mathscr {P}^{\left( t \right) }$$,*Pr*).

The probability of reconstruction is controlled by parameter *Pr*, and the reconstruction of the *t*th iteration is as Algorithm 1. The time complexity of the Algorithm 1 is dominated by the random selection and slicing steps. For each pair of individuals, the slicing and reconstruction operations take constant time. Since the process needs to be repeated for at most $\lceil \frac{Pr \times n}{2} \rceil$ individuals, the time complexity is $O(n)$, where $n$ represents the population size.

#### Augmentation of co-clustering results

Augmentation of co-clustering results refers to randomly selecting certain individuals in the *t*th generation population $\mathscr {P}^{\left( t \right) }$ and changing certain elements within the individuals according to defined rules, also known as perturbation. By adding random perturbations to individuals in $\mathscr {P}^{\left( t \right) }$ to increase its diversity, and then, the population can further avoid falling into local optima during the iteration process. Specifically, in cases where $\mathscr {P}^{\left( t \right) }$ contains a significant number of identical individuals, the reconstruction operation discussed previously will have difficulty obtaining new individuals, and in such cases the addition of the augmentation of co-clustering results addresses this challenge and ensures the consistent and stable introduction of new individuals to $\mathscr {P}^{\left( t \right) }$.

The probability of augmentation is controlled by parameter *Pa*, and the reconstruction of the *t*th iteration is as Algorithm 2, where $u\in \left[ 1,N \right] ,\ v\in \left[ N+1,N+M \right] ,\alpha _u\in \left[ 1,k_r \right] , \beta _v\in \left[ 1,k_c \right] ,u,v,\alpha _u,\,\,\beta _v\in \mathbb {Z}^+$,and $k_r$ and $k_c$ represent the number of sample clusters and feature clusters within dataset $\textbf{X}$, respectively.

**Algorithm 2:**
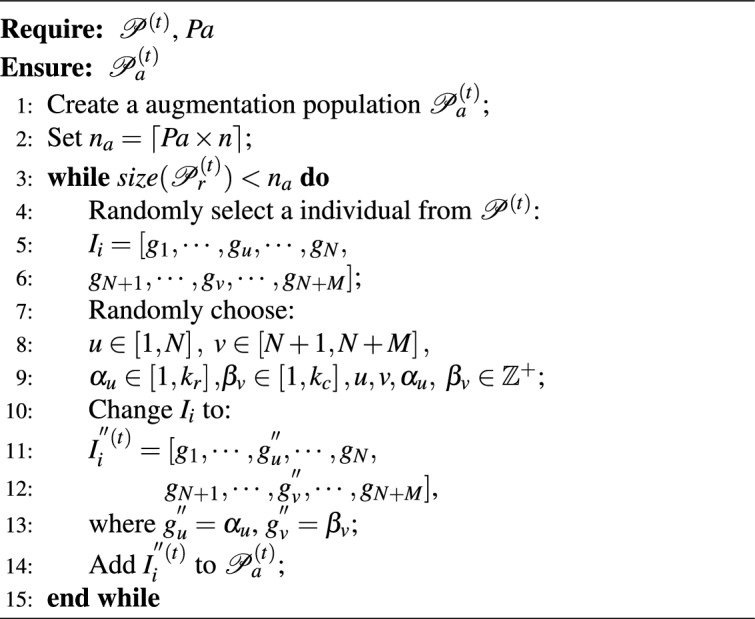
Augmentation($$\mathscr {P}^{\left( t \right) }$$,*Pa*).

The time complexity of the Algorithm 2 is primarily determined by the random selection and modification of each individual in the population. For each individual, changing the cluster labels takes constant time, and since this operation is performed for $\lceil Pa \times n \rceil$ individuals, the overall time complexity is $O(n)$, where $n$ is the population size.

#### Optimization of co-clustering results

Clearly, in the *t*th round of operations, we can obtain three populations of the *t*th generation: $\mathscr {P}^{\left( t \right) }$, $\mathscr {P}_{a}^{\left( t \right) }$ and $\mathscr {P}_{r}^{\left( t \right) }$. Notably, $\mathscr {P}_{a}^{\left( t \right) }$ and $\mathscr {P}_{r}^{\left( t \right) }$are derived from $\mathscr {P}^{\left( t \right) }$ through specific operations of Reconstruction and Augmentation, respectively. And then, by merging the three populations of the *t*th generation, we obtain population $\mathbb {P}^{\left( t \right) }=\mathscr {P}^{\left( t \right) }\cup \mathscr {P}_{a}^{\left( t \right) }\cup \mathscr {P}_{r}^{\left( t \right) }$, and $\ \left| \mathbb {P}^{\left( t \right) } \right|>n$. Based on this, the optimization of co-clustering results refers to the process of selecting *n* individuals from population $\mathbb {P}^{\left( t \right) }$ to form the population of the $(t+1)$th generation $\mathscr {P}^{\left( t+1 \right) }$. Then $\mathscr {P}^{\left( t+1 \right) }$ will decide whether to carry out the next iteration according to the established rules. the Optimization process is divided into two parts.

**A. Non-dominated Sorting of co-clustering results**The 4-dimensional fitness $\varGamma _i=\left[ \gamma _{1i},\,\,\gamma _{2i},\gamma _{3i},\gamma _{4i} \right] ^T$ of all $I_i$ in the set $\mathbb {P}^{\left( t \right) }$ is first calculated using Eq.(6). And then the fast non-dominated sorting approach^69^ is used to allocate the corresponding levels($F^{t}_1$, $F^{t}_2$ and so on) to all individuals in population $\mathbb {P}^{\left( t \right) }$.The processing steps are as Algorithm 3

$S_i$ is constructed for each co-clustering result $I_i$ in $\mathbb {P}^{\left( t \right) }$ to represent the other solutions dominated by $I_i$, the non-domination rank $k = 1$ is initialized, and $p_i$ is the count of solutions that dominate $I_i$. For any two distinct solutions $I_i$ and $I_j$ in $\mathbb {P}^{(t)}$, if for all $e \in \{1,2,3,4\}$, $\gamma _e(I_i) \le \gamma _e(I_j)$, then $I_i$ dominates $I_j$. The solution $I_i$ in $\mathbb {P}^{(t)}$ with $p_i = 0$ is populated into the set $F_k$. For each solution $I_i \in F_k$, subtracting the value of $p_j$ by 1, if $I_j \in S_i$. Incrementing *k* by 1 for the subsequent level of dominance. The process is repeated until all solutions in $\mathbb {P}^{(t)}$ have been populated into a non-dominated set.

**Algorithm 3:**
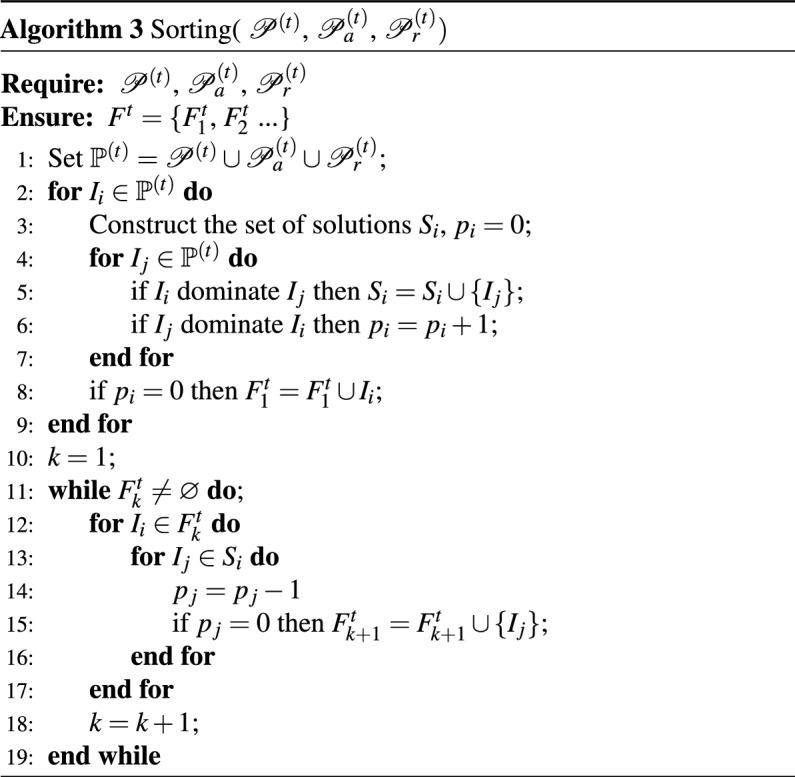
Sorting($$\mathscr {P}^{\left( t \right) }$$, $$\mathscr {P}_{a}^{\left( t \right) }$$, $$\mathscr {P}_{r}^{\left( t \right) })$$.

**Fig. 2:**
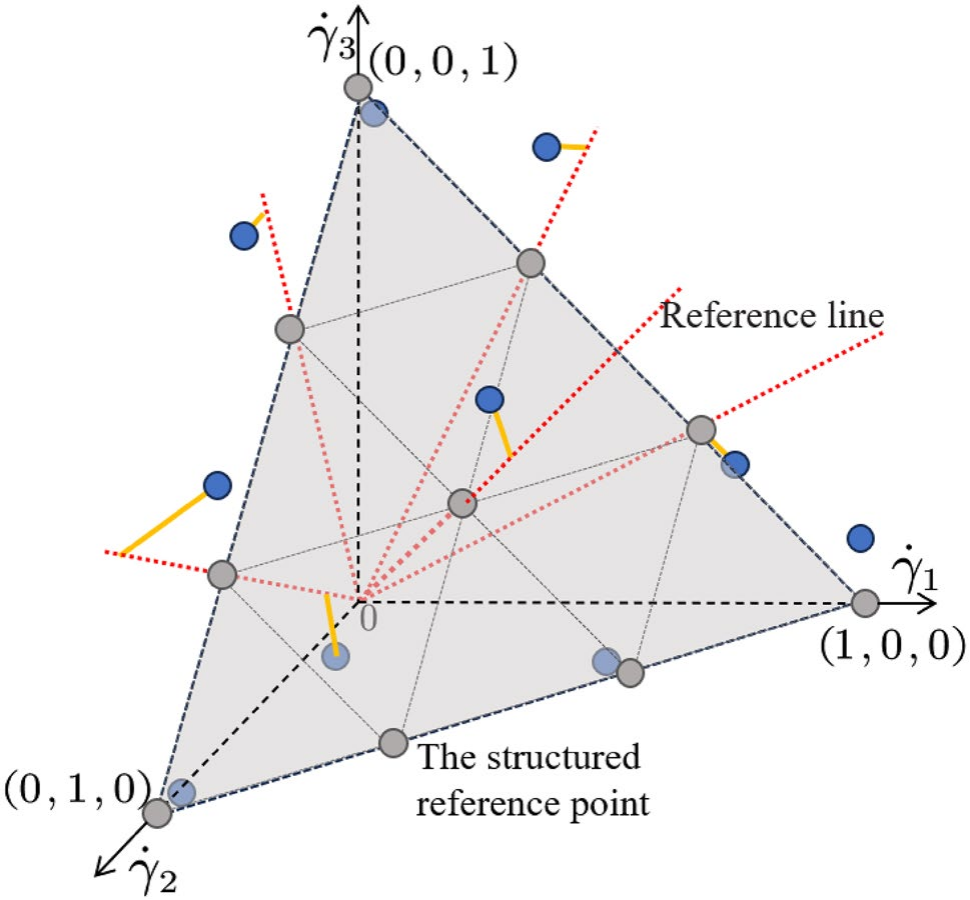
10 reference points are shown on a normalized reference plane for a three-objective problem with $\varrho =3$.The blue points represent the normalized solutions. The gray points represent the structured reference point ${z}^{re}\in \mathscr {Z}^{re}$. The red dashed line is the reference line corresponding to point ${z}^{re}$, and the yellow line indicates the connection between solution and the reference line(point).

**B. Non-dominated rank internal selection based on reference point** The solutions in the set $\mathbb {P}^{\left( t \right) }$ are assigned different non-dominated ranks by the part A operation, and the set of different ranks is filled in order into the new solution set $\mathscr {P}^{\left( t+1 \right) }$. When $\left| \mathscr {P}^{\left( t+1 \right) }\cup F^{t}_l \right|>\left| \mathscr {P}^{\left( t\right) }\right|$, it is necessary to perform a quadratic ordering in rank $F_k$ and pick some solutions to fill the set $\mathscr {P}^{\left( t+1 \right) }$ to satisfy $\left| \mathscr {P}^{\left( t+1 \right) } \right| =\left| \mathscr {P}^{\left( t \right) } \right|$. Since the object to be sorted has four evaluation metrics (as shown in Eq.(6)), we use a sorting method with a reference point to preserve the diversity of solutions^70^. An example is shown in Fig2, and the procedure is described as Algorithm 4.

Fistly, the minimum value $\overline{\gamma }=[ \gamma _{i=1:4}^{\min } ]^{T}$ of the objective function in $H_l=\cup _{\iota =1}^{l}F^{t}_{\iota }$ is found, where $l=\hat{\iota }:\left| \cup _{\iota =1}^{\hat{\iota }}F_{\iota }^{t} \right|>N>\left| \cup _{\iota =1}^{\hat{\iota }-1}F_{\iota }^{t} \right|$. Then, translating the function from Eq.(6) to

13 $$\begin{aligned} \begin{aligned} \ \varGamma ^{'}\left( I \right)= & \left[ \gamma _{1}^{'}\left( I \right) ,\ \gamma _{2}^{'}\left( I \right) ,\ \gamma _{3}^{'}\left( I \right) ,\ \gamma _{4}^{'}\left( I \right) \ \right] ^{T}, \end{aligned} \end{aligned}$$

where $$\gamma _{i}^{'}\left( I \right) =\gamma _{i}\left( I \right) -\gamma _{i}^{\min }$$.

Then, the extreme points $I^{\max }_i$ of each dimension is found by using the achievement scalarizing function.

14 $$\begin{aligned} \begin{aligned} I^{\max }_i&=I:\ argmin\,\,ASF\left( I,\,\,\omega _i \right) ,\\ ASF(I)&=\max _{i = 1}^{M}(\gamma ^{'}_{i}(I)/{\omega _i}),\ I\in {H_l}, \end{aligned} \end{aligned}$$

The weight vector $\omega _i = (\varepsilon _{1},\varepsilon _{2},\cdots ,\varepsilon _{M})$ is used to fix the target direction. When calculating the extreme point on the *i*th dimensional target axis, $\varepsilon _{i}=1$ and the other elements $\varepsilon _{j\ne i}=10e{-6}$.

Subsequently, the intercepts $[\hbar _{i=1:4}]$ of the hyper-plane determined by the extreme point set $\mathbb {I}^{max}=\{I_{i=1:4}^{\max }\}$ are calculated, and the normalized objective function group $\dot{\varGamma }\left( I \right) =[\dot{\gamma }_{i=1:4}\left( I \right) ]^{T}$ are obtained by Eq.(15).

15 $$\begin{aligned} \dot{\gamma }_i\left( I \right) =\frac{\gamma _i\left( I \right) -\gamma _{i}^{\min }}{{\hbar }_i-\gamma _{i}^{\min }} \end{aligned}$$

As shown in the Fig.2, the structured reference points $\mathscr {Z}^{re}$ are determined by the number of partitions $\varrho$. The number of reference points *H* is

16 $$\begin{aligned} H=\left| \mathscr {Z}^{re} \right| =\left( {\begin{array}{c}m+\varrho -1\\ \varrho \end{array}}\right) , \end{aligned}$$

where *m* represents the dimension of the objective function group.

Then, multiple reference lines are constructed by connecting each reference point to the origin. The normalized solution in the set $\cup _{\iota =0}^{l}F^{t}_{\iota }$ is connected to its shortest-distance(*d*(*I*)) reference line, denoted as $I: \pi (I)=z^{re}_j, I\in \cup _{\iota =0}^{l}F^{t}_{\iota },\ z^{re}_j\in \mathscr {Z}^{re}$, and $\rho _j$ is the number of solutions that are connected to $z^{re}_j$.

Finally, as individuals from set $\cup _{\iota =1}^{l-1}F^{t}_{\iota }$ have already been successfully transferred to $\mathscr {P}^{\left( t+1 \right) }$, the remaining task is to select $\left| \mathscr {P}^{\left( t \right) } \right| -\left| \cup _{\iota =1}^{l-1}F^{t}_{\iota } \right|$ solutions from the $F^{t}_l$ set to complete the filling of the next generation solution set $\mathscr {P}^{\left( t+1 \right) }$. During this filling process, maintaining the diversity of the solution set is crucial. To achieve this, it is necessary to give priority to solutions from the $F^{t}_l$ set that are connected with reference points of lower connectivity, as this helps preserve the diversity and breadth of the solution set. The complete procedure is presented in Algorithm 4.

**Algorithm 4:**
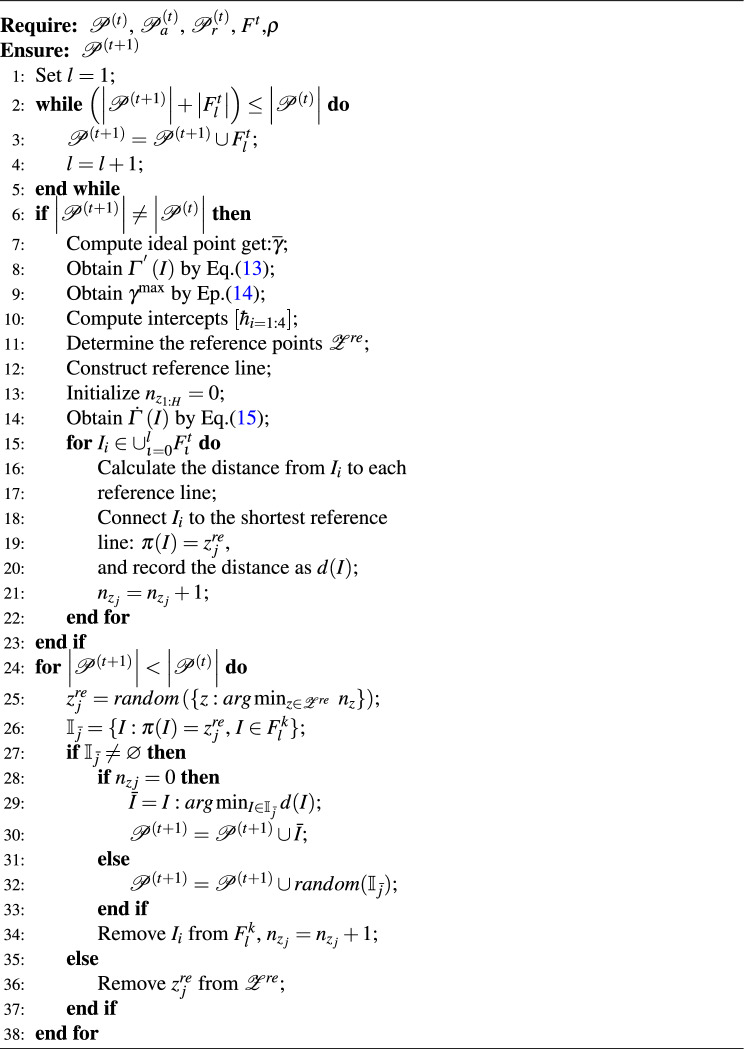
Selection($$\mathscr {P}^{\left( t \right) }$$, $$\mathscr {P}_{a}^{\left( t \right) }$$, $$\mathscr {P}_{r}^{\left( t \right) }$$, $$F^{t}$$,$$\varrho$$).

The overall time complexity of the optimization process is dominated by the non-dominated sorting and rank-based selection steps, which each have a complexity of $O(n^2 m)$. Thus, the overall time complexity of the optimization process is $O(n^2 m)$ per iteration, where $n$ is the population size and $m$ is the number of objectives.

#### Best selection of co-clustering results

After reaching the termination condition of optimization iteration, we select an optimal solution from the set $F^{T}_1$ that has the highest non-dominated rank. This selection is based on

17 $$\begin{aligned} \begin{aligned} cIT\left( I \right) =\sum _{i=1}^{k_r}{\sum _{j=1}^{k_c}{\sum _{\mathring{x}\in \mathbf {\mathring{X}}^{ij}}{\Vert \mathring{x}-\mu _{ij} \Vert }}}, \end{aligned} \end{aligned}$$

where $\mathbf {\mathring{X}}^{ij}=\textbf{X}\left[ label_r=i,\ label_c=j \right]$. Eq.(17) is used to evaluate the compactness of co-clustering. The smaller the value, the better the result of co-clustering. The values of $\text {label}_r$ and $\text {label}_c$ are derived from the solution $I$, and they are explicitly defined in Fig.1.

The time complexity of computing $cIT(I)$ for each individual is $O(k_r \cdot k_c \cdot N)$, where $k_r$ is the number of row clusters, $k_c$ is the number of column clusters, and $N$ is the number of samples. After calculating $cIT(I)$ for all individuals, we perform a sorting operation to select the optimal solution. The time complexity of sorting is $O(n \log n)$, where $n$ is the population size. Thus, the total time complexity for selecting the optimal co-clustering result is:${\O }(n \cdot (k_r \cdot k_c \cdot N) + n \log n)$

### Algorithm design

Based on the above detailed solution process for the objective function group, we summarize the entire SNSC workflow in the algorithm 5.

**Algorithm 5:**
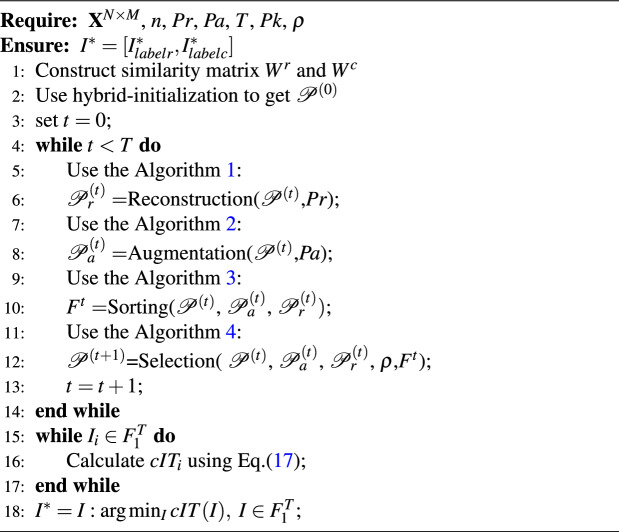
SNSC Algorithm.

### Time complexity of SNSC algorithm

The time complexity of constructing the sample similarity information in the SNSC algorithm is primarily composed of calculating the global and local similarity matrices. Specifically, computing the sample similarity matrix $\textbf{W}^{r1}$ involves pairwise distance calculations, which results in a complexity of $O(N^2 \cdot M)$. The calculation of the sample’s $\sigma ^r_i$ values requires determining the nearest neighbors for each sample, resulting in $O(N^2 \cdot n^{k}_r \cdot M)$. Similarly, the local sample similarity matrix $\textbf{W}^{r2}$ has a complexity of $O(N^2 \cdot M)$. Additionally, calculating the feature similarity matrix $\textbf{W}^{c}$ adds $O(M^2 \cdot N)$ complexity. The total time complexity for constructing the similarity information is therefore $O(N^2 \cdot M + N^2 \cdot n^{k}_r \cdot M + M^2 \cdot N + N \cdot n^{k}_l \cdot M^2)$.

The time complexity for the $T$ iterations of the SNSC algorithm is determined by several factors, including the objective function computations and sorting steps. For each iteration, the computations of the four objective functions for all individuals in the population have a complexity of $O(n \cdot (NM + k_r^2 M + k_c^2 N + N^2 + M^2))$, where $n$ is the population size and $k_r$ and $k_c$ are the number of row and column clusters, respectively. The non-dominated sorting step has a time complexity of $O(n^2 m)$, where $m$ is the number of objective functions. Considering all the steps in a single iteration, the overall time complexity is $O(T \cdot (n \cdot (NM + k_r^2 M + k_c^2 N + N^2 + M^2) + n^2 m))$, where $T$ is the number of iterations.

The time complexity for selecting the best co-clustering solution is dominated by the need to evaluate the compactness of co-clustering results using Eq. $17$, which has a complexity of $O(n \cdot (k_r \cdot k_c \cdot N))$. Additionally, sorting the solutions based on their non-dominated rank adds a complexity of $O(n \log n)$. Thus, the total complexity for the best selection of co-clustering results is $O(n \cdot (k_r \cdot k_c \cdot N) + n \log n)$.

In summary, the overall time complexity of the entire algorithm, considering $T$ iterations, is given by:

$$O\left( n \cdot (N + M) + T \cdot \left[ n \cdot (NM + k_r^2 M + k_c^2 N + N^2 + M^2) + n^2 m \right] + n \cdot (k_r \cdot k_c \cdot N) + n \log n \right) .$$

This expression accounts for the time spent on hybrid initialization, objective function computations, sorting operations, and the best selection of co-clustering results.

## Experiment design and result analysis

### Datasets and evaluation indexes

Experiments were conducted on 12 real datasets selected from the Microsoft Research Asia Multimedia (MSRA) and University of California Irvine (UCI) machine learning databases to test the effectiveness of different algorithms. The detailed information of the datasets are listed in Table 1.

**Table 1:** Datasets.

ID	Dataset	sample number	feature number	categories
D1	breast	683	9	2
D2	ionosphereEW	351	34	2
D3	amber	880	892	3
D4	balloon	830	892	3
D5	beverage	873	892	3
D6	ambulances	930	892	3
D7	breakfast	895	892	3
D8	birthdaycake	932	892	3
D9	anonovo	732	892	3
D10	banana	840	892	3
D11	airplane	855	892	3
D12	seeds	210	7	3
D13	internet advertisements-1	3279	1558	2
D14	secom	1567	590	2

Both external and internal indicators are considered in this section. Four evaluation indicators are used to measure the performance of co-clustering algorithms. Moreover, the statistical test is used to scientifically prove the superiority of the SNSC.

Clustering Accuracy (ACC) is a classical external evaluation index used to test the quality of clustering results and its definition is

18 $$\begin{aligned} ACC = \frac{\sum _{i=1}^{N}\delta (g_i,map(\hat{g_i}))}{N}. \end{aligned}$$

In the Eq.(18), *N* is the number of the samples of in the dataset. When $g_i=map(\hat{g_i})$, the value of $\delta (g_i,map(\hat{g_i}))$ is 1. In other cases, the value of $\delta (g_i,map(\hat{g_i}))$ is 0. $g_i$ and $\hat{g_i}$ are the real cluster labels and predicted cluster labels of sample $x_i$, respectively, and $map(\hat{g_i}))$ is a function that maps predicted labels to true labels.

ACC is chosen due to its simplicity and ability to provide a clear measure of clustering performance. In co-clustering tasks, where both rows (samples) and columns (features) are clustered simultaneously, ACC effectively quantifies the accuracy of the clustering results, directly reflecting how well the model assigns correct labels. This metric is crucial for evaluating the alignment between the predicted and true co-clusters, ensuring that the proposed method captures the underlying structure of the data.

The total cluster sum of square(TSSE) is a common internal indicator used to evaluate the effectiveness of clustering results, and better the clustering result with a smaller value of *TSSE*. In order to better evaluate the effect of co-clustering results, we make small changes to TSSE to get cTSSE. TSSE is defined as

19 $$\begin{aligned} TSSE = \sum _{j = 1}^{k_r}SSE_{j}, \end{aligned}$$

where $SSE_{j}$ represents the square error criterion of the jth row cluster, which is defined as

20 $$\begin{aligned} SSE_{j} = \sum _{x_i \in R_j}{(x_i - \mu ^r_j)^2}. \end{aligned}$$

$\mu ^r_j$ is the cluster center of the *j*th row cluster $R_j$, which calculates the sum of the squares of the distances from each data sample in the *j*th row cluster to its cluster center. Obviously, the smaller the value of TSSE, the better the clustering results.

In order to better evaluate the results of co-clustering, we use cTSSE as the final internal evaluation index, which is defined as

21 $$\begin{aligned} cTSSE = \sum _{i=1}^{k_r}\sum _{j=1}^{k_c}cSSE_{ij}{,} \end{aligned}$$

where $k_r$ is the number of row clusters, and $k_c$ is the number of column clusters. $cSSE_{ij}$ is the sum of the square of the distance from the data in the block cluster composed of the *i*th row cluster $\mathscr {R}_i$ and the *j*th row cluster $\mathscr {C}_j$ to its nearest cluster center, specifically. $cSSE_{ij}$ is defined as

22 $$\begin{aligned} cSSE_{ij} = \sum _{p:x_p \in \mathscr {R}_i}\sum _{q:f_q \in \mathscr {C}_j}{(e_{pq} - \mu _{ij})^2} \end{aligned}$$

where $e_{pf}$ is the data sequence of the sample $x_p$ in the row cluster $\mathscr {R}_i$ and the feature column $f_q$ in the column cluster $\mathscr {C}_j$. Since the final computed results may be quite large, we performed a normalization step on $cSSE_{ij}$ in this step to ensure more stable and consistent values during the optimization process. According to the definition, it can be known that the smaller the value of cTSSE, the better the cohesion of the samples in the cluster, and the better the effect of co-clustering. Thus, cTSSE is a more appropriate internal evaluation metric for co-clustering, as it captures the interactions between rows and columns, which are critical to the quality of the co-clustering results.

The Adjusted Rand Index (ARI) is a widely used external evaluation metric that measures the similarity between two clustering results while correcting for chance. Unlike metrics like Clustering Accuracy (ACC), which directly compares predicted labels with true labels, ARI accounts for the inherent randomness in clustering and provides a more robust evaluation of clustering performance. This makes ARI particularly valuable when the number of clusters or the nature of the clustering problem may vary across datasets.

ARI is defined as:

23 $$\begin{aligned} \text {ARI} = \frac{\text {RI} - \mathbb {E}[\text {RI}]}{\max (\text {RI}) - \mathbb {E}[\text {RI}]}, \end{aligned}$$

where $\text {RI}$ is the Rand Index, and $\mathbb {E}[\text {RI}]$ represents the expected value of the Rand Index under random clustering. The ARI ranges from −1 to 1, where 1 indicates perfect agreement between the predicted and true clustering results, 0 indicates random clustering, and negative values indicate worse than random clustering. ARI is less biased towards non-uniform and non-balanced clustering structures, making it more appropriate for datasets with varying cluster sizes or imbalanced distributions.

Normalized Mutual Information (NMI) is an external evaluation metric that measures the amount of shared information between the predicted and true clusterings, normalized by the entropy of both clusterings. NMI is defined as:

24 $$\begin{aligned} \text {NMI}(U, V) = \frac{I(U, V)}{\sqrt{H(U)H(V)}}, \end{aligned}$$

where $I(U, V)$ is the mutual information between the predicted and true clusters, and $H(U)$ and $H(V)$ are the entropies of the predicted and true clustering results, respectively. NMI was included in this study due to its ability to evaluate the alignment between the predicted and true clusters in a manner that is less biased towards non-uniform and non-balanced clustering structures.

In this paper, a large number of comparative algorithms are used to construct comparative experiments to verify the performance of the proposed algorithm. Although the above two evaluation indexes consider the external validity index and internal validity index of clustering respectively to evaluate the performance of the algorithm, in order to distinguish the difference between the proposed algorithm and the comparison algorithm more intuitively, we introduce the relevant theories of statistical test Friedman test and Iman-Davenport to measure the difference between the algorithms. The use of this indicator can be summarized in three steps.

Firstly, the algorithm is sorted according to the evaluation index. The algorithm with the best performance ranks first, the algorithm with the second performance ranks second, and so on.

Secondly, we calculate the Friedman statistic, assuming that we compare the differences of $n^a$ algorithms on $N^{data}$ datasets. The definition of *Friedman* statistic is $\tau _{\chi ^2}=\frac{12N^{data}}{n^a\left( n^a+1 \right) }\left( \sum _{i=1}^{n^a}{r}_{i}^{2}-\frac{n^a\left( n^a+1 \right) ^2}{4} \right)$ where $r_i$ refers to the ranking value of the *i*th algorithm.

Finally, the variable $\tau _{F}$ is calculated. On the basis of Friedman statistics, Iman and Davenport proposed the variable $\tau _{F}$, which is defined as

25 $$\begin{aligned} \tau _{F}=\frac{(N^{data}-1)\tau _{\chi ^2}}{N^{data}{(n^a-1)}-\tau _{\chi ^2}}. \end{aligned}$$

In subsequent experiments, we will use Eq.(25) to measure the performance differences between different algorithms.

### Parameter setting and comparison algorithm

In this experimental setup, the parameters are defined as Population size is established at $n=70$, reconstruction probability at $Pr=0.7$, augmentation probability at $Pa=0.3$, Partition number is $\varrho =4$, and the cap for the maximum number of iterations is set at $T=150$. Additionally, the neighbor parameter *Pk* is assigned a value of 0.1.

During the hybrid-initialization phase, a variety of learners, such as Fuzzy C-means^82^, *k*-means^80^, *k*-medoids^83^, and Gaussian Mixed Model^84^, are employed to extract self-supervised information.

While the SNSC algorithm leverages unsupervised methods to gather supervised information, it fundamentally remains categorized as an unsupervised co-clustering algorithm. Therefore, the five comparative algorithms selected for this experiment are derived from both classical and cutting-edge unsupervised co-clustering methodologies(ITCC^35^, DRCC^18^, NMFAN^51^, SNCC^17^, TRNMTF^19^, BCOT^21^ and PB-$\tau$CC^22^). This selection aims to provide a comprehensive evaluation of SNSC’s capabilities within the spectrum of unsupervised co-clustering approaches.

### Comparative analysis of experimental results

In this section, we provide a detailed comparative analysis of the experimental results to evaluate the performance of our proposed SNSC model. The analysis is approached from two main angles: first, by comparing SNSC with other state-of-the-art co-clustering algorithms, and second, by assessing the impact of different initialization strategies on SNSC’s performance. This comprehensive comparison aims to provide insights into both the effectiveness and efficiency of our model.

#### Comparison with other algorithms

**Table 2:** Comparison of ACC.

Dataset	SNSC	ITCC	DRCC	NMFAN	SNCC	TRNMTFs	BCOT	PB-$\tau$CC
D1	0.9640(2.0)	0.7533(6.0)	0.6751(7.0)	0.9559(3.0)	0.5476(8.0)	**0.9701(1.0)**	0.9531(4.0)	0.8785(5.0)
D2	**0.7009(1.0)**	0.6211(7.5)	0.6402(5.0)	0.6353(6.0)	0.6211(7.5)	0.6558(3.0)	0.6695(2.0)	0.6467(4.0)
D3	0.5870(3.0)	0.5472(4.0)	**0.6143(1.0)**	0.5207(6.0)	0.5455(5.0)	0.4659(7.0)	0.4602(8.0)	0.5920(2.0)
D4	**0.5551(1.0)**	0.4158(6.0)	0.4546(3.0)	0.4989(2.0)	0.3940(7.0)	0.4346(4.0)	0.3711(8.0)	0.4217(5.0)
D5	**0.5557(1.0)**	0.4058(7.0)	0.4107(5.0)	0.4906(2.0)	0.4066(6.0)	0.4038(8.0)	0.4513(4.0)	0.4880(3.0)
D6	**0.6232(1.0)**	0.4705(5.0)	0.4722(4.0)	0.5406(2.0)	0.4344(8.0)	0.4451(7.0)	0.4559(6.0)	0.5065(3.0)
D7	**0.6019(1.0)**	0.4659(8.0)	0.5145(5.0)	0.4985(7.0)	0.5162(3.5)	0.5220(2.0)	0.5162(3.5)	0.4994(6.0)
D8	**0.6092(1.0)**	0.4932(6.0)	0.5004(5.0)	0.5040(4.0)	0.5043(3.0)	0.5379(2.0)	0.4045(8.0)	0.4710(7.0)
D9	0.4429(6.0)	0.4631(3.0)	0.4555(5.0)	0.4053(8.0)	**0.4795(1.0)**	0.4605(4.0)	0.4672(2.0)	0.4372(7.0)
D10	0.4333(5.0)	**0.4749(1.0)**	0.4698(2.0)	0.4101(6.0)	0.4357(4.0)	0.3974(7.0)	0.4571(3.0)	0.3952(8.0)
D11	**0.4691(1.0)**	0.4358(3.0)	0.4405(2.0)	0.4200(4.0)	0.3953(6.0)	0.3827(8.0)	0.3883(7.0)	0.3988(5.0)
D12	0.6190(8.0)	0.8429(3.0)	0.6471(6.0)	0.7143(5.0)	0.6381(7.0)	0.7200(4.0)	0.8857(2.0)	**0.9286(1.0)**
D13	0.6290(2.0)	0.0161(8.0)	0.5484(4.5)	**0.8710(1.0)**	0.5323(6.0)	0.5645(3.0)	0.5484(4.5)	0.5161(7.0)
D14	0.7569(3.0)	0.0006(8.0)	0.7147(5.0)	**0.9336(1.0)**	0.7396(4.0)	0.8245(2.0)	0.6075(7.0)	0.6822(6.0)
*Ave*	**0.6105**	0.4576	0.5399	0.5999	0.5136	0.5561	0.5454	0.5616
$Ave_r$	**2.5714**	5.3929	4.25	4.0714	5.4286	4.4286	4.9286	4.9286

Table 2 and Table 3 present the comparative analysis of ACC and cTSSE metrics across 14 datasets utilizing the SNSC, ITCC, DRCC, NMFAN, SNCC, TRNMTF, BCOT and PB-$\tau$CC algorithms. Each value in the table represents the average result of 10 runs. Optimal values for each dataset are underscored, while bracketed figures following each metric denote the algorithm’s performance rank on that particular dataset, and the last two rows(*Ave* and $Ave_r$) of table 2 and table 3 are the average evaluation index values and the average values of rank, respectively. A detailed examination of Tables 2 and 3 follows.

**Table 3:** Comparison of cTSSE.

Dataset	SNSC	ITCC	DRCC	NMFAN	SNCC	TRNMTFs	BCOT	PB-$\tau$CC
D1	7.6793(5.0)	10.4814(8.0)	7.9842(6.0)	6.4099(2.0)	8.0763(7.0)	**6.1428(1.0)**	6.5151(3.0)	7.2275(4.0)
D2	**6.3636(1.0)**	7.5670(4.0)	7.5832(5.0)	8.3117(7.0)	7.6750(6.0)	7.2517(2.0)	7.3092(3.0)	8.9946(8.0)
D3	**3.7029(1.0)**	5.0693(5.0)	4.7912(3.0)	5.2790(8.0)	5.2234(6.0)	5.2240(7.0)	4.7838(2.0)	4.9863(4.0)
D4	**4.6262(1.0)**	5.5872(5.0)	5.3096(3.0)	5.6197(6.0)	5.7160(7.0)	5.0758(2.0)	5.4547(4.0)	5.8064(8.0)
D5	**4.2965(1.0)**	4.9615(6.0)	4.6415(3.0)	5.0158(7.0)	5.0750(8.0)	4.7259(4.0)	4.5815(2.0)	4.8214(5.0)
D6	**3.3238(1.0)**	4.5620(7.0)	4.2070(2.0)	4.4689(5.0)	4.7033(8.0)	4.2997(3.0)	4.4551(4.0)	4.5130(6.0)
D7	**2.9033(1.0)**	4.5802(8.0)	4.4604(5.0)	4.3071(4.0)	4.5401(7.0)	4.2586(3.0)	4.4664(6.0)	4.2052(2.0)
D8	4.1925(2.0)	5.1583(7.0)	4.8021(4.0)	4.9801(5.0)	5.1256(6.0)	**3.8955(1.0)**	5.1607(8.0)	4.7223(3.0)
D9	**4.1745(1.0)**	5.4295(8.0)	5.2724(4.0)	5.3697(5.0)	5.4150(7.0)	5.1725(3.0)	5.3802(6.0)	4.7513(2.0)
D10	**3.6364(1.0)**	5.1060(7.0)	4.8048(3.0)	5.2241(8.0)	5.0557(6.0)	4.5771(2.0)	4.8540(4.0)	4.8854(5.0)
D11	**1.6291(1.0)**	2.7173(5.0)	2.6396(3.0)	2.6658(4.0)	3.0001(6.0)	2.5355(2.0)	4.8181(8.0)	3.9136(7.0)
D12	3.2853(7.0)	3.4573(8.0)	2.7830(6.0)	2.4911(4.0)	2.5564(5.0)	2.2115(3.0)	**1.9625(1.0)**	1.9841(2.0)
D13	86.4251	**0.0000**	90.9423	87.5037	90.6124	91.9604	79.0841	74.0795
D14	7736.2726(2.0)	**3038.4646(1.0)**	9161.8321(5.0)	8228.4857(4.0)	7779.0345(3.0)	11239.3464(8.0)	9768.3705(7.0)	9605.3234(6.0)
*Ave*	598.9297	**221.6530**	709.3162	637.5868	603.169	868.8244	756.0086	743.5488
$Ave_r$	**2.0714**	5.7143	4.2143	5.2857	6.2857	3.5	4.3571	4.5714

From Table 2, the SNSC algorithm demonstrates superior ACC values on 14 datasets compared to its counterparts. The average ACC value for SNSC across all 12 datasets stands at 0.6105, surpassing the average scores of ITCC(0.4576), DRCC(0.5399), NMFAN(0.5999), SNCC(0.5136), TRNMTF(0.5561), BCOT(0.5454) and PB-$\tau$CC(0.5616). This equates to SNSC’s average ACC outperforming the comparative algorithms by $33.41\%$, $13.08\%$, $1.77\%$, $18.87\%$, $9.78\%$, $11.93\%$ and $8.71\%$ respectively. Additionally, the average ranking for SNSC in terms of the ACC index across the 14 datasets is the highest, indicating its superior average performance across the board.

The hypothesis test for the comparison of algorithms is conducted using the Friedman test. The Friedman statistic ($\widetilde{\tau }_{\chi ^2}$) is calculated as follows:

26 $$\begin{aligned} \begin{array}{c} {\widetilde{\tau }_{\chi ^2}}= \frac{12\times {14}}{8\times (8+1)}(2.5714^2+5.3929^2+ 4.25^2+4.0714^2 + 5.4286^2+ 4.4286^2+4.9286^2\\ +4.9286^2-\frac{8\times {(8+1)^2}}{4}) \approx {13.9940}. \end{array} \end{aligned}$$

Then the $\tau _{F}$ variable is calculated as

27 $$\begin{aligned} \tau _{F}=\frac{(14-1)\tau _{\chi ^2}}{14\times {(8-1)}-\tau _{\chi ^2}}\approx {2.1656}. \end{aligned}$$

Given that $\tau _{F}$ follows an *F*-distribution with degrees of freedom (7) and (91), the p-value is obtained from the *F*-distribution, yielding a p-value of 0.0445. Based on this result, we reject the null hypothesis at the 0.05 significance level, confirming that the performance of the proposed SNSC algorithm is significantly different from the performance of the comparative algorithms.

Secondly, as shown in Table 3, the SNSC algorithm has better performance on datasets D2, D3, D4, D5, D6, D7, D9, D10, and D11. In other words, the cTSSE value obtained by the SNSC algorithm is better than the cTSSE value of the comparison algorithm on other datasets except datasets D1, D8, D12,D13 and D14.

It is worth noting that the average cTSSE value obtained by the SNSC algorithm across 9 datasets is the smallest (the smaller the better the performance of the algorithm). This performance is statistically significant, as evidenced by the results of the hypothesis test. The Friedman statistic ($\widetilde{\tau }_{\chi ^2}$) was calculated to be 32.8974, and Iman’s statistic ($\tau _F$) was computed as 8.3750. With degrees of freedom 7 and 84, the corresponding p-value obtained from the F-distribution is $3.538 \times 10^{-5}$, which is far below the 0.05 significance level. This p-value indicates that the performance of the SNSC algorithm is statistically superior to its counterparts, further confirming its outstanding performance under the cTSSE metric.

**Table 4:** Comparison of ARI.

Dataset	SNSC	ITCC	DRCC	NMFAN	SNCC	TRNMTFs	BCOT	PB-$\tau$CC
D1	0.8575(3.0)	0.0111(8.0)	0.1059(7.0)	**0.8741(1.5)**	0.1919(6.0)	**0.8741(1.5)**	0.8193(4.0)	0.5723(5.0)
D2	**0.1587(1.0)**	0.0223(7.0)	0.0788(5.0)	0.0833(4.0)	0.0526(6.0)	0.0836(3.0)	0.1116(2.0)	0.0180(8.0)
D3	0.1802(2.0)	0.0000(7.0)	**0.1864(1.0)**	0.0000(7.0)	0.0000(7.0)	0.0535(4.0)	0.1109(3.0)	0.0430(5.0)
D4	0.0891(2.0)	0.0000(5.0)	0.0535(3.0)	0.0000(5.0)	0.0000(5.0)	**0.1039(1.0)**	−0.0248(7.0)	−0.0488(8.0)
D5	0.0126(5.0)	0.0000(7.5)	0.0421(3.0)	0.0000(7.5)	0.0015(6.0)	**0.1008(1.0)**	0.0364(4.0)	0.0444(2.0)
D6	0.0236(2.0)	0.0000(6.0)	0.0089(3.0)	0.0000(6.0)	0.0000(6.0)	**0.1607(1.0)**	−0.0023(8.0)	0.0008(4.0)
D7	0.0340(5.0)	0.0000(7.5)	**0.1452(2.0)**	0.0000(7.5)	0.0312(6.0)	0.1487(1.0)	0.1042(3.0)	0.0716(4.0)
D8	0.0021(5.0)	0.0000(7.0)	**0.1133(2.0)**	0.0000(7.0)	0.0000(7.0)	0.1458(1.0)	0.0226(4.0)	0.0800(3.0)
D9	0.0137(5.0)	0.0000(7.0)	0.0707(2.0)	0.0000(7.0)	0.0000(7.0)	**0.0426(3.0)**	0.0772(1.0)	0.0345(4.0)
D10	0.0225(3.0)	0.0000(7.0)	**0.0412(1.0)**	0.0000(7.0)	0.0000(7.0)	0.0002(5.0)	0.0278(2.0)	0.0121(4.0)
D11	0.0241(3.0)	0.0000(6.0)	0.0364(2.0)	0.0000(6.0)	0.0000(6.0)	0.0057(4.0)	**0.0406(1.0)**	−0.0059(8.0)
D12	0.4805(6.0)	0.0000(8.0)	0.2990(7.0)	0.7364(2.0)	0.6045(5.0)	0.6300(4.0)	0.6998(3.0)	**0.8002(1.0)**
D13	−0.0144(7.0)	0.0000(3.0)	−0.0058(4.5)	**0.5433(1.0)**	−0.0113(6.0)	0.0017(2.0)	−0.0058(4.5)	−0.0160(8.0)
D14	0.0054(2.0)	0.0000(6.5)	**0.0084(1.0)**	0.0000(6.5)	0.0019(4.0)	0.0044(3.0)	0.0017(5.0)	−0.0061(8.0)
*Ave*	0.1350	0.0024	0.0846	0.1598	0.0623	**0.1683**	0.1442	0.1143
$Ave_r$	3.6429	6.6071	3.1071	5.3571	6.0000	**2.4643**	3.6786	5.1429

**Table 5:** Comparison of NMI.

Dataset	SNSC	ITCC	DRCC	NMFAN	SNCC	TRNMTFs	BCOT	PB-$\tau$CC
D1	0.7614(3.0)	0.3391(6.0)	0.1238(8.0)	**0.7825(1.5)**	0.2181(7.0)	**0.7825(1.5)**	0.7158(4.0)	0.5655(5.0)
D2	0.1195(2.0)	**0.3476(1.0)**	0.0501(5.0)	0.0473(6.0)	0.0279(7.0)	0.0651(4.0)	0.0770(3.0)	0.0118(8.0)
D3	0.1258(4.0)	**0.3542(1.0)**	0.1539(2.0)	0.0000(7.5)	0.0000(7.5)	0.0335(5.0)	0.1260(3.0)	0.0282(6.0)
D4	0.0572(2.0)	**0.3803(1.0)**	0.0454(4.0)	0.0000(7.5)	0.0000(7.5)	0.0475(3.0)	0.0186(5.0)	0.0161(6.0)
D5	0.0045(7.0)	**0.3611(1.0)**	0.0398(3.0)	0.0000(8.0)	0.0100(6.0)	0.0450(2.0)	0.0291(4.0)	0.0249(5.0)
D6	0.0105(3.0)	**0.3514(1.0)**	0.0064(5.0)	0.0000(7.5)	0.0000(7.5)	0.0612(2.0)	0.0080(4.0)	0.0015(6.0)
D7	0.0197(7.0)	**0.3734(1.0)**	0.1070(2.0)	0.0000(8.0)	0.0278(6.0)	0.0821(3.0)	0.0714(4.0)	0.0532(5.0)
D8	0.0254(6.0)	**0.3681(1.0)**	0.1177(2.0)	0.0000(7.5)	0.0000(7.5)	0.0915(3.0)	0.0551(5.0)	0.0751(4.0)
D9	0.0272(6.0)	**0.4023(1.0)**	0.0647(2.0)	0.0000(7.5)	0.0000(7.5)	0.0587(4.0)	0.0639(3.0)	0.0500(5.0)
D10	0.0347(4.0)	**0.4002(1.0)**	0.0474(2.0)	0.0000(7.5)	0.0000(7.5)	0.0092(6.0)	0.0379(3.0)	0.0161(5.0)
D11	0.0253(4.0)	**0.3960(1.0)**	0.0351(3.0)	0.0000(7.5)	0.0000(7.5)	0.0045(5.5)	0.0534(2.0)	0.0045(5.5)
D12	0.5720(6.0)	0.4533(7.0)	0.3646(8.0)	0.6932(3.0)	0.5928(5.0)	0.6795(4.0)	0.7029(2.0)	**0.7611(1.0)**
D13	0.0308(3.0)	0.3970(2.0)	0.0092(5.5)	**0.4837(1.0)**	0.0057(7.0)	0.0136(4.0)	0.0092(5.5)	0.0002(8.0)
D14	0.0001(5.0)	**0.1822(1.0)**	0.0005(2.0)	0.0000(7.5)	0.0000(7.5)	0.0001(5.0)	0.0001(5.0)	**0.0003(3.0)**
*Ave*	0.1296	**0.3647**	0.0833	0.1433	0.0630	0.1410	0.1406	0.1149
$Ave_r$	4.4286	**1.8571**	3.8214	6.2500	7.0000	3.7143	3.7500	5.1786

Tables 4, 5, and 6 present the results of eight algorithms across the ARI, NMI, and runtime metrics. In terms of ARI and NMI, the SNSC algorithm demonstrates a moderate performance, with average rankings of 3.6429 and 4.4286 in the ARI and NMI tables, respectively. While SNSC does not lead in these specific metrics, it still maintains a competitive position. In contrast, when evaluating runtime, SNSC ranks sixth, performing only better than BCOT and PB-$\tau$CC, indicating that its computational cost is relatively higher compared to other algorithms.

Despite its moderate performance in ARI and NMI, SNSC excels in other critical areas such as ACC and cTSSE. Hypothesis testing further confirms that SNSC holds a clear advantage over its counterparts in these performance indicators, securing its absolute leading position in these aspects. This demonstrates that SNSC, although not the fastest, offers superior clustering quality, particularly in terms of accuracy and stability.

In conclusion, while SNSC demonstrates solid performance in ACC and cTSSE, its performance in ARI and NMI remains moderate, with average rankings of 3.6429 and 4.4286, respectively. The runtime comparison highlights areas where SNSC could be optimized for better efficiency. These insights provide a clear direction for future improvements, where balancing the high clustering quality with reduced computational cost could further elevate SNSC’s performance across all metrics. Therefore, SNSC remains a strong candidate for clustering tasks where quality and robustness are prioritized, even though optimization for efficiency remains an area for future work.

**Table 6:** Comparison of runtime.

Dataset	SNSC	ITCC	DRCC	NMFAN	SNCC	TRNMTFs	BCOT	PB-$\tau$CC
D1	260.4452(8.0)	0.7402(2.0)	**0.1215(1.0)**	1.8333(4.0)	0.7699(3.0)	2.6615(5.0)	3.7010(6.0)	3.9354(7.0)
D2	50.0247(8.0)	1.3071(4.0)	**0.0265(1.0)**	0.5025(3.0)	0.3872(2.0)	2.6534(5.0)	6.5355(7.0)	6.2865(6.0)
D3	1412.5415(5.0)	1724.1787(6.0)	**0.3911(1.0)**	5.5585(3.0)	2.0551(2.0)	12.7376(4.0)	8620.8935(8.0)	8109.9851(7.0)
D4	1342.7009(5.0)	1613.6237(6.0)	**0.4299(1.0)**	5.5829(3.0)	5.3476(2.0)	14.5177(4.0)	8068.1185(8.0)	7590.3871(7.0)
D5	1489.8785(5.0)	1757.4886(6.0)	**0.4410(1.0)**	5.4025(2.0)	5.5498(3.0)	14.8068(4.0)	8787.4430(8.0)	8267.1385(7.0)
D6	1629.6445(5.0)	2696.2932(6.0)	**0.4333(1.0)**	6.9929(3.0)	2.2081(2.0)	17.8811(4.0)	13481.4660(8.0)	12680.1714(7.0)
D7	1720.2536(5.0)	2495.3261(6.0)	**0.4078(1.0)**	6.8646(3.0)	2.4389(2.0)	17.5531(4.0)	12476.6305(8.0)	11735.2868(7.0)
D8	1766.6294(5.0)	2498.4896(6.0)	**0.3633(1.0)**	4.6895(3.0)	1.7381(2.0)	10.2085(4.0)	12492.4480(8.0)	11750.0737(7.0)
D9	1079.1171(6.0)	1021.1003(5.0)	**0.3137(1.0)**	4.0455(2.0)	4.6885(3.0)	8.6566(4.0)	5105.5015(8.0)	4804.2721(7.0)
D10	1257.0788(5.0)	1300.9777(6.0)	**0.3734(1.0)**	4.2387(3.0)	1.3039(2.0)	9.4001(4.0)	6504.8885(8.0)	6120.6478(7.0)
D11	1294.6246(6.0)	1230.0677(5.0)	**0.3285(1.0)**	4.3117(3.0)	1.3224(2.0)	10.1060(4.0)	6150.3385(**8.0**)	5787.6188(7.0)
D12	24.4451(8.0)	0.0437(2.0)	**0.0145(1.0)**	0.1849(3.0)	0.4805(6.0)	2.6780(7.0)	0.2185(4.0)	0.2600(5.0)
D13	2180.6850(8.0)	77.7545(5.0)	**0.4590(1.0)**	15.0937(4.0)	1.9884(2.0)	2.2521(3.0)	388.7725(7.0)	369.2564(6.0)
D14	2287.9817(6.0)	1724.5787(5.0)	**0.7067(1.0)**	14.9253(4.0)	2.4968(2.0)	12.3146(3.0)	8622.8935(8.0)	8115.6316(7.0)
*Ave*	1271.1465	1295.8550	**0.3436**	5.7305	2.3411	9.8877	6479.2749	6095.7822
$Ave_r$	6.0714	5.0000	**1.0000**	3.0714	2.5000	4.2143	7.4286	6.7143

#### Comparison of different initialization methods

The impact of different initialization strategies on the performance of the SNSC model is investigated. The original SNSC model, which uses Hybrid-initialization, is compared with two variations: SNSCr, which uses random-initialization, and SNSCh, which employs heuristic-initialization. The goal is to assess how these initialization methods affect clustering quality and the overall performance of SNSC, providing insights into the role of initialization in achieving optimal clustering results.

**Table 7:** Comparison of performance metrics: ACC and cTSSE for different initialization strategies.

Indexes	ACC			cTSSE		
Data	SNSC	SNSCr	SNSCh	SNSC	SNSCr	SNSCh
**D1**	0.9640(3.0)	**0.9663(1.0)**	0.9649(2.0)	7.6793(3.0)	6.4478(2.0)	**5.2194(1.0)**
**D2**	**0.7009(1.0)**	0.6980(2.0)	0.6841(3.0)	6.3636(3.0)	**5.4472(1.5)**	**5.4472(1.5)**
**D3**	**0.5870(1.0)**	0.4841(2.0)	0.4216(3.0)	**3.7029(1.0)**	4.3614(2.0)	4.4001(3.0)
**D4**	0.5551(2.0)	0.4663(3.0)	**0.5759(1.0)**	**4.6262(1.0)**	5.0323(3.0)	4.7398(2.0)
**D5**	**0.5557(1.0)**	0.5223(3.0)	0.5441(2.0)	**4.2965(1.0)**	4.3312(2.0)	4.3857(3.0)
**D6**	**0.6232(1.0)**	0.5000(3.0)	0.5011(2.0)	**3.3238(1.0)**	3.8810(3.0)	3.8652(2.0)
**D7**	**0.6019(1.0)**	0.6011(2.5)	0.6011(2.5)	**2.9033(1.0)**	5.1269(3.0)	5.0791(2.0)
**D8**	0.6092(2.0)	**0.6170(1.0)**	0.4378(3.0)	4.1925(2.0)	**4.0374(1.0)**	4.4661(3.0)
**D9**	0.4429(3.0)	**0.5219(1.5)**	**0.5219(1.5)**	**4.1745(1.0)**	4.9043(3.0)	4.2043(2.0)
**D10**	**0.4333(1.0)**	0.4226(2.0)	0.4214(3.0)	3.6364(2.0)	**3.5446(1.0)**	4.2414(3.0)
**D11**	**0.4691(1.0)**	0.4058(3.0)	0.4585(2.0)	**1.6291(1.0)**	4.1633(3.0)	3.1239(2.0)
**D12**	0.6190(3.0)	**0.6571(1.5)**	**0.6571(1.5)**	3.2853(2.0)	3.2853(2.0)	3.2853(2.0)
**Ave**	**0.5968**	0.5719	0.5658	**4.1511**	4.5469	4.3715
$Ave_r$	**1.6667**	2.1250	**2.2083**	**1.5833**	2.2083	2.2083

**Table 8:** Comparison of performance metrics: ARI and NMI for different initialization strategies.

Indexes	ARI			NMI		
Data	SNSC	SNSCr	SNSCh	SNSC	SNSCr	SNSCh
**D1**	0.8575(3.0)	**0.8685(1.0)**	0.8630(2.0)	0.1195(2.0)	0.1195(2.0)	0.1195(2.0)
**D2**	0.1587(2.0)	0.1587(2.0)	0.1587(2.0)	0.1258(2.0)	0.1258(2.0)	0.1258(2.0)
**D3**	**0.1802(1.0)**	−0.0011(3.0)	0.0036(2.0)	0.0572(2.0)	**0.1265(1.0)**	0.0209(3.0)
**D4**	**0.0891(1.0)**	0.0877(2.0)	0.0000(3.0)	0.0045(2.0)	**0.0566(1.0)**	0.0000(3.0)
**D5**	0.0126(2.0)	0.0083(3.0)	**0.0264(1.0)**	0.0105(2.0)	0.0045(3.0)	**0.0237(1.0)**
**D6**	**0.0236(1.0)**	−0.0087(2.0)	−0.0157(3.0)	**0.0197(1.0)**	0.0015(3.0)	0.0032(2.0)
**D7**	**0.0340(1.0)**	−0.0013(2.5)	−0.0013(2.5)	**0.0254(1.0)**	0.0108(2.0)	0.0062(3.0)
**D8**	0.0021(2.0)	**0.1334(1.0)**	−0.0128(3.0)	0.0272(2.0)	**0.1212(1.0)**	0.0099(3.0)
**D9**	0.0137(3.0)	0.0798(2.0)	**0.0891(1.0))**	0.0347(2.0)	0.0347(2.0)	0.0347(2.0)
**D10**	**0.0225(1.0)**	0.0201(2.0)	0.0181(3.0)	0.0253(3.0)	**0.0304(1.0)**	0.0277(2.0)
**D11**	**0.0241(1.0)**	0.0233(2.0)	0.0000(3.0)	**0.5720(1.0)**	0.0244(2.0)	0.0000(3.0)
**D12**	0.4805(2.0)	0.4805(2.0)	0.4805(2.0)	0.0308(2.0)	0.0308(2.0)	0.0308(2.0)
**Ave**	**0.1582**	0.1541	0.1341	**0.0877**	0.0572	0.0335
$Ave_r$	**1.6667**	2.0417	2.2917	**1.8333**	1.8333	2.3330

**Fig. 3:**
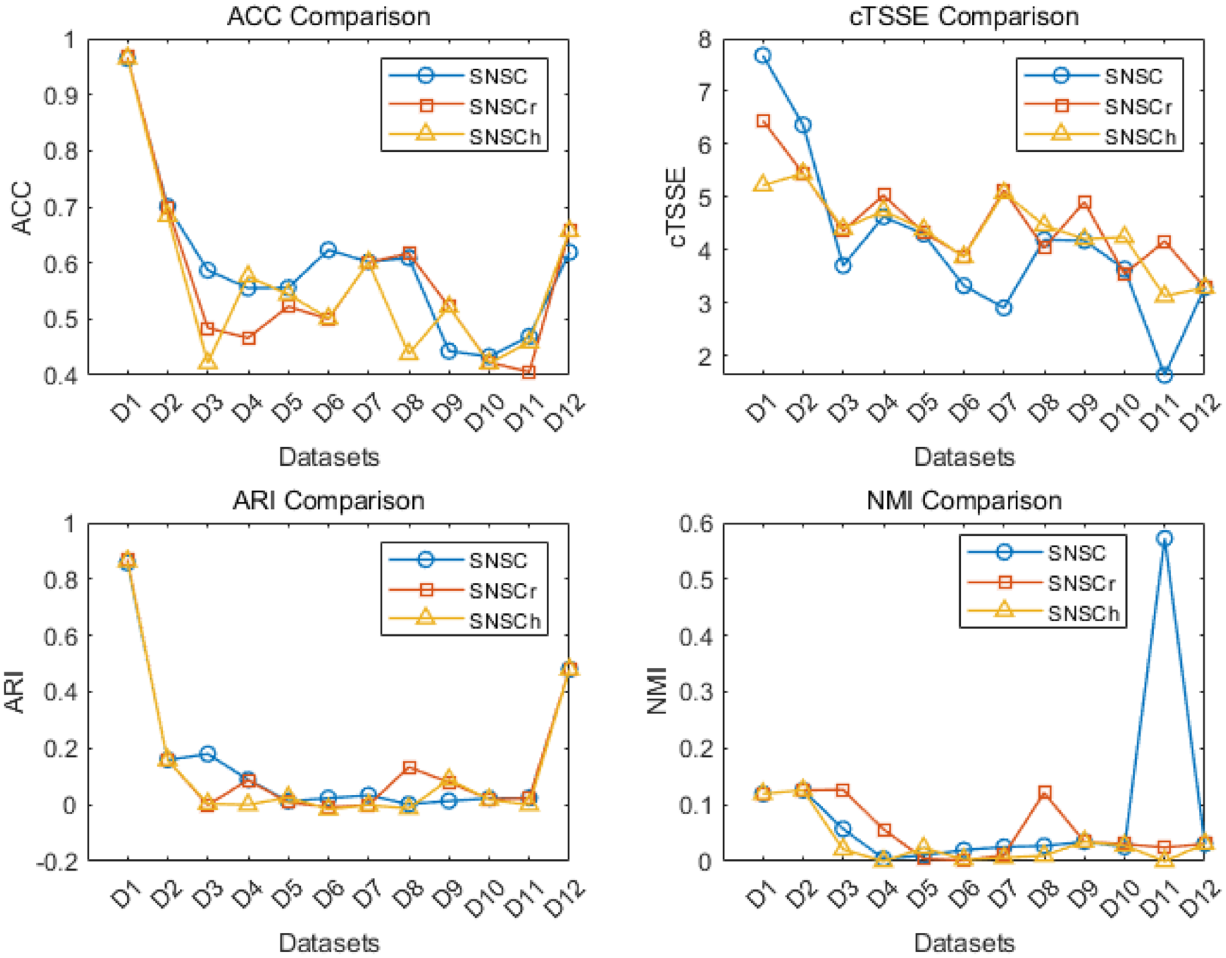
Performance Comparison of SNSC, SNSCr, and SNSCh Across Different Datasets: ACC, cTSSE, ARI, and NMI Indexes.

As shown in Tables 7 and 8, the SNSC algorithm outperforms both SNSCr and SNSCh on average across 9 datasets, demonstrating that the chosen hybrid initialization scheme is effective. When only heuristic initialization is used, although a good solution is found early in the iterations, the search capability for new solutions diminishes as the solutions in the set become increasingly homogeneous over time. On the other hand, when only random initialization is used, the randomness and associated uncertainty significantly hinder the algorithm’s ability to find optimal solutions. In conclusion, the hybrid initialization scheme employed by SNSC proves to be effective. For more intuitive results, please refer to Fig. 3.

## Conclusion

In this paper, the Self-supervised Non-dominated Sorted Model for Co-clustering (SNSC) is introduced as a novel approach to address the challenges of co-clustering. The SNSC model employs a multi-objective optimization strategy, which aligns seamlessly with the multi-objective nature of co-clustering tasks. By leveraging supervisory information from the original data, we enhance data utilization and significantly improve the performance of SNSC, while preserving its unsupervised framework. Comprehensive experiments confirm the superior performance of SNSC across various datasets.

However, the SNSC model does have some limitations that should be addressed for broader applicability. One key limitation is the computational complexity of the algorithm, especially when applied to large-scale datasets. The SNSC model relies on a genetic optimization algorithm, which typically benefits from a higher number of iterations and a larger population size. These factors can lead to better optimization results. However, as the iteration count and population size increase, so does the computational burden. From the experimental runtime results, it is evident that the algorithm’s time complexity is relatively high, which can hinder its efficiency when applied to large-scale data. In particular, the time required for processing large datasets may be a significant drawback in real-time or time-sensitive applications.

In light of these limitations, further work should focus on improving the algorithm’s scalability and reducing its time complexity. Potential approaches include exploring more efficient genetic algorithms, reducing the population size or iteration count without sacrificing optimization performance, or incorporating parallel computing techniques to enhance processing efficiency. Additionally, adaptive parameter tuning strategies could be investigated to minimize the sensitivity to parameter settings, making the model more flexible and efficient for large-scale datasets.

## Data Availability

The datasets used and/or analysed during the current study available from the corresponding author on reasonable request.
